# Co-designing eHealth and Equity Solutions: Application of the Ophelia (Optimizing Health Literacy and Access) Process

**DOI:** 10.3389/fpubh.2020.604401

**Published:** 2020-11-20

**Authors:** Christina Cheng, Gerald R. Elsworth, Richard H. Osborne

**Affiliations:** ^1^Centre for Global Health and Equity, Faculty of Health, Arts and Design, Swinburne University of Technology, Hawthorn, VIC, Australia; ^2^School of Health and Social Development, Faculty of Health, Deakin University, Burwood, VIC, Australia

**Keywords:** co-design, eHealth literacy, health equity, digital divide, digital health, ophelia process, eHLQ

## Abstract

**Background:** The unequal access, challenges and outcomes related to using technology have created the digital divide, which leads to health inequalities. The aim of this study was to apply the Ophelia (Optimizing Health Literacy and Access) process, a widely used systematic approach to whole of community co-design, to the digital context to generate solutions to improve health and equity outcomes.

**Methods:** This was a mixed method study. A cross-sectional survey was undertaken at 3 health organizations in Victoria, Australia using the eHealth Literacy Questionnaire (eHLQ) as a needs assessment tool. Cluster analysis was conducted to identify subgroups with varying eHealth literacy needs. These data, combined with semi-structured interviews with clients, were used to generate vignettes representing different eHealth literacy profiles. The vignettes were presented at co-design workshops with clients and health professionals to generate solutions for digital health services improvement. Expert validation and proof-of-concept testing was explored through mapping the process against Ophelia guiding principles.

**Results:** The cluster analyses identified 8 to 9 clusters with different profiles of eHealth literacy needs, with 4 to 6 vignettes developed to represent the eHealth literacy strengths and weaknesses of clients at each of the 3 sites. A total of 32, 43, and 32 solutions across 10 strategies were co-created based on ideas grounded in local expertise and experiences. Apart from digital solutions, non-digital solutions were frequently recommended as a strategy to address eHealth literacy needs. Expert validation identified at least half of the ideas were very important and feasible, while most of the guiding principles of the Ophelia process were successfully applied.

**Conclusion:** By harnessing collective creativity through co-design, the Ophelia process has been shown to assist the development of solutions with the potential to improve health and equity outcomes in the digital context. Implementation of the solutions is needed to provide further evidence of the impact of the process. The suggested inclusion of non-digital solutions revealed through the co-design process reminds health organizations and policymakers that solutions should be flexible enough to suit individual needs. As such, taking a co-design approach to digital health initiatives will assist in preventing the widening of health inequalities.

## Introduction

Technological advancement has ushered in a new frontier for health care delivery on a personal level. From seeking information to making appointments, monitoring health to managing health records, eHealth or digital health has revolutionized how health information and services are accessed and used in recent years (1–3). In the wake of the COVID-19 pandemic, telehealth has become an important tool in providing patient consultations and treatments during lockdown. The health care industry describes the pandemic as a “breakthrough event” for digital health and expects the acceptance and usage of digital health solutions will continue to increase (4). The World Health Organization (WHO) also acknowledges that digital technologies have the potential to play a major role in improving public health and recommends prioritizing development and use of health technologies to advance the health-related aims of the Sustainable Development Goals (5). However, not everyone has the same access or skills to take advantage of the benefits and convenience of digital health.

The unequal access, challenges and outcomes related to using technology have created a gap between users and non-users or unskilled users, described as the digital divide (6–9), leading to the potential widening of health inequalities in the age of eHealth (10, 11). The WHO also cautions that innovation and technology should be used to help reduce inequities instead of becoming another mechanism for leaving people behind (12).

Studies of the digital divide relating to health found that barriers faced by the digitally disadvantaged populations can be linked to low eHealth literacy (13–15), defined as “the ability to seek, find, understand, and appraise health information from electronic sources and apply the knowledge gained to addressing or solving a health problem” (16). For any eHealth or digital health solutions to be adopted, it is posited that the eHealth literacy needs of users must be addressed (16–18). Besides, it is recommended that user-centered principles, with requirements of users as the primary focus (19), be applied in digital health intervention development (20, 21). In line with the user-centered principles is the co-design approach (22). It has been advocated that patients and the workforce should take a more direct and active role in identifying, implementing and evaluating health care solutions (22–24). Robert et al. even argue that patients are indeed the biggest resources for quality of care improvement (24). This approach uses the lived experience of users for service design, with users as active advisors and consultants (19). It is described as “collective creativity as it is applied across the whole span of a design process” (22), and the approach has been found to develop a sense of ownership among users (25–27). Co-design is considered best practice in research for indigenous people in Australia, Canada, and New Zealand and the power-sharing between developers and users can serve “to reduce inequality and empower vulnerable communities” (28).

Yet, in a recent systematic review of eHealth interventions targeted at socially disadvantaged groups who are most at risk of low eHealth literacy, it was found that user-centered principles were not discussed, and eHealth literacy needs were generally not considered. User involvements were usually in the form of focus groups for needs assessment which involved limited respondents or at usability testing when the intervention was already designed (29). The findings reflect the growing concern that there is a lack of frameworks or guidelines to inform the development of digital health solutions that meet eHealth literacy needs (30), and disadvantaged populations are overlooked in digital health solution design (31). As such, vulnerable groups are at risk of becoming marginalized in the digital age (29, 32).

With a co-design approach as one of the guiding principles, the Ophelia (Optimizing Health Literacy and Access) process is a method for co-creating solutions to improve access, equity and outcomes by addressing health literacy needs (25, 26). Studies have found considerable success in using the Ophelia process to co-design intervention ideas, using the Health Literacy Questionnaire (HLQ) as an assessment tool. In systematically applying the process to 9 health sites in Victoria, Australia, 21–78 intervention ideas within each of the sites were generated (26). Another application in a Melbourne public hospital setting produced 15 potential solutions across 3 key themes for the improvement of hospital care and services (33). A cardiac rehabilitation setting in Denmark also applied the Ophelia process and generated 47 unique ideas to improve the unit's health literacy responsiveness (34). Using intervention ideas generated from the Ophelia process, BreastScreen Victoria of Australia has recorded significantly increase in the number of screening bookings among women of culturally and linguistically diverse backgrounds (35). There are also other Ophelia projects currently underway in several European countries as part of the WHO National Health Literacy Demonstration Projects (34). However, to date, the Ophelia process has not been used to develop digital health solutions.

The aim of this study was to determine if the Ophelia process can be adapted into the digital context and applied to co-design solutions addressing eHealth literacy needs. By harnessing local wisdom from users and stakeholders as recommended in the process, it was expected that ideas grounded in local experience and expertise can lead to eHealth solutions for the improvement of health and equity outcomes.

## Materials and Methods

This was a mixed method study undertaken at Victoria, Australia from March 2018 to April 2019. Ethics approval was obtained from the Deakin University Human Research Ethics Committee (HEAG-H 146_2017).

### Settings and Respondents

Participating organizations included 3 health sites: (1) a private primary care medical practice (i.e. practice which clients may be required to pay additional consultation fees) in metropolitan Melbourne (Site 1); (2) a not-for-profit community health service in metropolitan Melbourne (Site 2); and (3) a private primary care medical practice in regional Victoria (Site 3). The inclusion criteria were: (1) aged 18 years or older; (2) with or without any health condition; and (3) able to complete the questionnaire in paper-based format, web-based format or face-to-face interview. Exclusion criteria were: (1) experiencing obvious cognitive or mental health issues; (2) clinically unwell as deemed by their treating health care professionals; and (3) insufficient English to complete the questionnaire and do not have a family member or carer to assist them.

### The Ophelia Process

The Ophelia process is built on the foundations of intervention mapping, quality improvement collaboratives and realist synthesis as previous described (25) and is guided by 8 principles described in Table 1.

**Table 1:** The 8 guiding principles of the Ophelia (Optimizing Health Literacy and Access) process^*^.

**Principle**	**Description**
P1. Outcomes focused	Improved health and reduced health inequities
P2. Equity driven	All activities at all stages prioritize disadvantaged groups and those experiencing inequity in access and outcome
P3. Co-design approach	In all activities at all stages, relevant stakeholders engage collaboratively to design solutions
P4. Needs-diagnostic approach	Participatory assessment of local needs using local data
P5. Driven by local wisdom	Intervention development and implementation is grounded in local experience and expertise
P6. Sustainable	Optimal health literacy practice becomes normal practice and policy
P7. Responsiveness	Recognize that health literacy needs and the appropriate responses vary across individuals, contexts, countries, cultures, and time
P8. Systematically applied	A multilevel approach in which resources, interventions, research and policy are organized to optimize health literacy

*P, Principle*.

* *Adapted from Beauchamp et al. Table 1, p. 5 (26)*.

**Figure 1:**
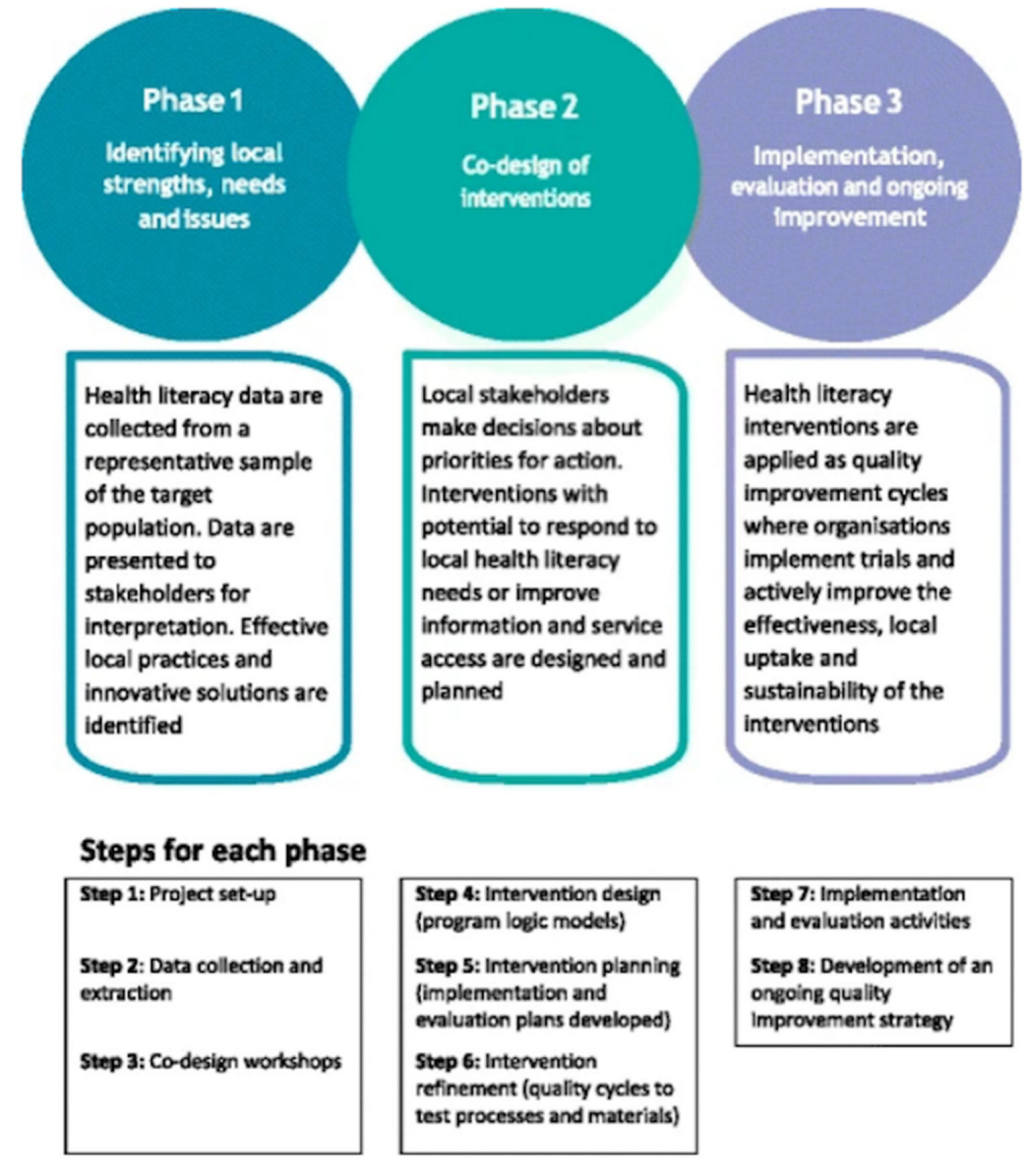
The Ophelia (Optimzing Health Literacy and Access) process. Source: Reproduced from Beauchamp et al. Figure 1, p. 5 (26).

The process involves 3 phases: (1) identifying needs; (2) co-design of interventions; and (3) implementation and evaluation (see Figure 1). Health literacy needs of target users are usually assessed by the HLQ, a multidimensional health literacy needs assessment tool (36). The HLQ was developed using a grounded, validity-driven approach aimed to assess people's experiences in understanding, accessing and using health information and services (36, 37). It has demonstrated strong construct validity and reliability in various contexts and settings (36, 38–40). The HLQ consists of 44 items representing 9 dimensions of health literacy: (1) Feeling understood and supported by health care providers; (2) Having sufficient information to manage my health; (3) Actively managing my health; (4) Social support for health; (5) Appraisal of health information; (6) Ability to actively engage with health care providers; (7) Navigating the healthcare system; (8) Ability to find good health information; and (9) Understanding health information well-enough to know what to do (36). The results from the HLQ needs assessment help create health literacy profiles which are then developed into vignettes, depicting the lived experience of people facing different health literacy challenges. These vignettes/stories are then presented at co-design (ideas generation) workshops with target users and frontline health professionals to harness local wisdom and generate solutions. These ideas are then acted upon based on organizational priorities and go through implementation, evaluation and ongoing improvement (25, 26).

The current study sought to undertake Phase 1 of the Ophelia process and the instrument for needs assessment was replaced with a questionnaire used for assessing eHealth literacy.

#### Step 1 – Project Set-Up

Three health organizations participated in the study seeking to understanding the eHealth literacy needs of their clients and generate ideas to improve health and equity outcomes at their organization.

#### Step 2 – Data Collection and Extraction

This step involved using the eHealth Literacy Questionnaire (eHLQ) as the needs assessment tool. Cluster analysis was then conducted to identify subgroups with varying eHealth literacy needs. The results were combined with semi-structure interviews to develop vignettes to be presented at the co-design workshops at Step 3.

##### The eHealth literacy questionnaire (eHLQ)

The multidimensional eHealth Literacy Questionnaire (eHLQ) was used rather than the eHealth Literacy Scale (eHEALS) (30, 41) because the eHEALS has a limited focus on information searching or evaluation (42) and is unsuitable for the cluster analysis and vignette development. The eHLQ was developed using a grounded, validity-driven approach (43). Through concept mapping workshops and an international online survey, the perspectives and experiences of a wide range of eHealth stakeholders including patients, health care providers, health informatic professionals, public health researchers and computer scientists, were integrated and 7 domains of eHealth literacy were identified:

(1) Using technology to process health information;

(2) Understanding of health concepts and language;

(3) Ability to actively engage with digital services;

(4) Feel safe and in control;

(5) Motivated to engage with digital services;

(6) Access to digital services that work; and

(7) Digital services that suit individual needs (44, 45).

Validity testing of the tool showed satisfactory evidence of construct validity and reliability across various settings (45). The eHLQ consists of 35 items with 7 scales representing the 7 dimensions of eHealth literacy. Each scale has 4–6 items, relating to a 4-point response option of strongly disagree, disagree, agree and strongly agree. Scale scores are calculated by averaging the item scores within each scale with equal weighting, each with a score range of 1–4 (45).

A cross-sectional survey was undertaken at the 3 health sites using the eHLQ. Recruitment of respondents was undertaken by approaching all clients present in the waiting area during set times at each site. A respondent information form was provided, and the completion of questionnaire was regarded as implied consent. Respondents were provided with the options of self-administration by paper or online or face-to-face interview. The interview option was provided so that people with likely low eHealth literacy could easily participate to maximize participation, equity, and quality of the research.

Additional demographic data including date of birth, sex, postcode, language spoken at home, education, health status, perceived health status, and use of technology were collected. Contact information of respondents was only collected if they indicated that they were interested in taking part in the semi-structured interviews and/or workshops.

##### Cluster analysis

Cluster analysis is an analytical method for examining multivariate data and identifying groups of homogeneous observations (46, 47). It has been advocated as a patient-tailored approach to provide better understanding of heterogeneity among patient groups to allow for personalized and efficient interventions (48–50). To ensure equity planning for interventions, the Ophelia process recommends the use of cluster analysis, based on the 7 scale scores of the eHLQ, to classify target users into groups with different sets of eHealth literacy strengths and limitations.

To perform a cluster analysis, different techniques can be undertaken and which method to use depends on the type of variables, the aim of the analysis as well as intuition of the researchers (46, 47, 51). The approach used in the Ophelia process is hierarchical cluster analysis using Ward's method for linkage (25). A total of 3–20 solutions were specified in the current study.

For the selection of an optimal clustering solution, 2 main criteria were used based on the Ophelia process. The first was to examine the standard deviation of the scores. A standard deviation of above 0.6 indicated considerable variation within the cluster, however, it should also be noted that standard deviation could be high for small clusters. The second criterion was to consider the demographic data linked to the clusters. Two groups with quite similar eHealth literacy profiles but different demographics might require different strategies. Hence, demographics of the clusters needs to be considered. While there are other recommendations such as ignoring extremely small clusters or using statistical tests to validate group differences (46, 47), these methods are not applicable to the Ophelia process. As the purpose of the analysis is to generate targeted solutions, small groups still deserve attention following the equity driven guiding principle of the Ophelia process (Table 1). Hence, each solution needs to be examined carefully for the final optimal solution.

There is no consensus on what constitutes an adequate sample size in cluster analysis to generate a stable solution (50). Given the 3 diverse settings, a minimum of 100 respondents was estimated for each site, based on the experience in other Ophelia settings (26, 33, 35, 52). The treatment of missing values involved excluding a scale if over 50% responses were missing for a certain respondent, in accordance with the HLQ scoring in the Ophelia process. Any respondent with one or more scale scores missing was excluded from the analysis (53). Data analysis was performed using IBM SPSS Version 25.0 (RRID:SCR_002865) (54). The selection of cluster solutions was initially undertaken by one researcher (CC) and then reviewed by and discussed with another researcher (RHO).

##### Semi-structured interview

The purpose of the semi-structured interview was to gain insight into people's experiences in using digital health and provide context for the vignettes (25, 26). Respondents were mainly from the cross-sectional survey who agreed to take part and provided contact information. Oral consent was obtained if they agreed to be interviewed.

Each semi-structured interview was conducted over the phone and took about 30 minutes, covering their experiences of using technology. There were also questions specific to the use of digital health, privacy and whether they had support to use technology. Based on the interviewee's eHLQ scores, further questions were asked why they scored low on some scales. Respondents were also encouraged to discuss any other personal experiences or express their views on using eHealth. Notes were taken during the interview while the interviews were audio-recorded with consent from the respondents.

The data from the interviews were anonymized and combined with the cluster analyses to develop vignettes depicting the various experiences of how people used health information and services.

#### Step 3 – Co-design Workshops

Each co-design workshop is a brainstorming session for respondents to respond to the needs expressed in the vignettes, and usually requires 2–3 hours with 6–12 respondents (55). For this study, 1 community member workshop and 1 frontline health professional workshop were held at each site. Community members were recruited from the cross-sectional survey where respondents had provided contact details or referred by their health services. Frontline health professionals were recruited by senior managers of the organizations. All respondents provided written consent.

Each workshop started with an introduction of the project and an overview of the eHealth literacy survey results. Then, vignettes were discussed using the following guiding questions: (1) Do you know someone like this person/recognize this person in your clients? (2) What are this person's main problems? (3) What could be done to improve this person's health? and (4) What could community organizations/your organization do if there are lots of people/clients like this person?

Thematic analysis of the ideas, using mainly an inductive approach based on the content, was undertaken for each site. The ideas were further categorized into four levels: individual, family, practitioner and policy levels as described in the Ophelia process (25, 55). The analyses were undertaken by 1 researcher (CC) and results were reviewed by and discussed with another researcher (RHO).

### Evidence for Application of the Ophelia Process

#### Expert Validation of Co-designed Solutions

Expert validation (56, 57) through experts (3 managers or staff of the participating sites) served as initial evidence of the potential usefulness of the solutions. Respondents were recruited by senior managers of the organizations. The solutions from the co-design workshops, thematically summarized, were organized into a questionnaire where the ideas were rated in terms of their importance and feasibility as well as to provide an estimation of the current situation. The rating was from 1 “not important at all” to 5 “essential,” similarly, feasibility was rated from 1 “not feasible at all” to 5 “highly feasible and can be fully implemented,” and current situation, was rated from 1 “never implemented” to 5 “fully implemented.”

#### Proof-of-Concept of Application

A proof-of-concept was defined as successful application of the 8 Ophelia guiding principles for an Ophelia project (26). The results of this study were mapped against the 8 guiding principles (Table 1), to determine how well the principles had been operationalized. The evaluation served as evidence for the feasibility of using the Ophelia process in the digital context.

## Results

### Respondents Characteristics and Overall eHealth Literacy

A total of 207, 206, and 117 questionnaires were collected at Site 1, Site 2, and Site 3, respectively. Respondents aged from 18 to 94, with about 60% of them being female. About one-third of the respondents had university education. At least one-third at Site 1 (33.3%) and Site 2 (40.8%) spoke a language other than English at home while Site 3 respondents were mostly English-speaking clients (92.3%). Site 2 had the highest proportion of respondents who reported having some form of long-term chronic health condition (65%), comparing to Site 1 (49.8%) and Site 3 (57.3%). Use of technology was the highest among Site 3 respondents with 80% used a computer or laptop while Site 1 had 72% and Site 2 only had 65%. The use of the internet for information was also the highest among Site 3 respondents (85%), comparing to Site 1 (75%) and Site 2 (68%). See Table 2 for details.

**Table 2:** Sociodemographic characteristics of respondents of Site 1 (metropolitan primary care medical practice), Site 2 (metropolitan community health), and Site 3 (regional primary care medical practice).

Characteristics*	**Site 1 (*n* = 207)**	**Site 2 (*n* = 206)**	**Site 3 (*n* = 117)**
	***n* (%)**	***n* (%)**	***n* (%)**
Age (mean, SD) years	53.1 (19.4)	61.4 (18.3)	55.1 (16.6)
	Range: 19–93	Range: 18–94	Range: 24–91
**Sex**			
Female	125 (60.4)	124 (60.2)	74 (63.2)
Male	80 (38.6)	82 (39.8)	43 (36.3)
**Education**			
Primary school or less	4 (1.9)	2 (1.0)	0 (0.0)
Completed primary school	9 (4.3)	11 (5.3)	1 (0.9)
Did not complete secondary school	20 (9.7)	31 (15.0)	18 (15.4)
Completed secondary school	40 (19.3)	42 (20.4)	26 (22.2)
TAFE^∧^/trade certificate/diploma	56 (27.1)	46 (22.3)	39 (33.3)
Completed university	74 (35.7)	69 (33.5)	33 (28.2)
**Language at home**			
English	137 (66.2)	122 (59.2)	108 (92.3)
Other	69 (33.3)	84 (40.8)	9 (7.7)
Socioeconomic status**			
IRSD 1 – 2 (lowest)	89 (43.0)	0 (0.0)	29 (24.8)
IRSD 3 – 4	2 (1.0)	1 (0.5)	3 (2.6)
IRSD 5 – 6	42 (20.3)	51 (24.8)	19 (16.2)
IRSD 7 – 8	66 (31.9)	69 (33.5)	0 (0.0)
IRSD 9 – 10 (highest)	2 (1.0)	76 (36.9)	63 (53.8)
**Private health insurance**			
Yes	116 (56.0)	75 (36.4)	60 (51.3)
No	89 (43.0)	124 (60.2)	57 (48.7)
**Longstanding illness (may have more than one)**			
None	105 (50.2)	72 (35.0)	50 (42.7)
Arthritis	29 (14.0)	58 (28.2)	31 (26.5)
Cancer	1 (0.5)	10 (4.9)	3 (2.6)
Heart disease	29 (14.0)	43 (20.9)	18 (15.4)
Diabetes	18 (8.7)	41 (19.9)	9 (7.7)
Respiratory condition	7 (3.4)	20 (9.7)	15 (12.8)
Anxiety	29 (14.0)	25 (12.1)	17 (14.5)
Depression	30 (14.5)	27 (13.1)	13 (11.1)
Other	31 (16.9)	38 (18.4)	19 (16.2)
**Perceived health status**			
Good to excellent	169 (83.7)	140 (68.0)	94 (80.3)
Fair to poor	34 (16.3)	58 (28.2)	23 (19.6)
**Ownership of digital device (may have more than one)**			
Computer/laptop	149 (72.0)	134 (65.0)	94 (80.3)
Mobile phone or smartphone	186 (91.6)	169 (84.9)	108 (92.3)
Tablet	100 (48.3)	85 (41.3)	58 (49.6)
Other	4 (1.9)	3 (1.5)	0 (0.0)
Average number of devices owned (mean, SD)	2.2 (0.9)	2.0 (1.0)	2.2 (0.8)
**Use of digital communication platform (may have more than one)**			
Email	155 (74.9)	141 (65.0)	102 (87.7)
Text message	159 (76.8)	139 (67.5)	102 (87.7)
Facebook	115 (55.6)	85 (41.3)	69 (59.0)
Twitter	14 (6.8)	10 (4.9)	7 (6.0)
Instagram	53 (25.6)	32 (15.5)	19 (16.2)
Snapchat	26 (12.6)	10 (4.9)	15 (12.8)
WhatsApp/WeChat	55 (26.6)	48 (23.3)	11 (9.4)
Blogging	5 (2.4)	5 (2.4)	5 (4.3)
Forum/chat room	10 (4.8)	9 (4.4)	7 (6.0)
Other	8 (3.9)	5 (2.4)	4 (3.4)
Number of platforms used (mean, SD)	3.0 (1.9)	2.4 (1.8)	2.9 (1.5)
Looked for online information	158 (76.3)	140 (68.0)	98 (83.8)
Monitored health digitally	68 (32.9)	70 (34.0)	45 (38.5)

SD, Standard deviation;

∧ TAFE, Technical and Further Education; *Characteristics presented as n (%) unless otherwise stated; **Socioeconomic status is classified by IRSD10 – The Index of Relative Socio-economic Disadvantage Decile 2016, ranking within Australia. This index is based on information provided by the Australian Bureau Statistics (58). Postcodes are divided into 10 ranks with higher number indicating more advantaged suburbs.

The overall eHealth literacy scores are presented in Table 3. The 3 sites demonstrated very similar scores. Most respondents appeared to have relatively good knowledge about their health conditions but might not always use technology for health. While they generally were comfortable with the security of eHealth systems, Site 2 respondents seemed to be not as confident as respondents of the other two sites. The scores also showed that many respondents from the 3 sites did not consider digital services met their needs.

**Table 3:** eHealth literacy scores of participants of Site 1, Site 2, and Site 3.

	**Scores (mean, SD)**		
	**Score range: 1 (low) – 4 (high)**		
**Scales**	**Site 1**	**Site 2**	**Site 3**
	**(*n* = 207)**	**(*n* = 206)**	**(*n* = 117)**
1. Using technology to process health information	2.56 (0.61)	2.57 (0.66)	2.66 (0.49)
2. Understanding of health concepts and language	2.92 (0.43)	2.96 (0.41)	2.92 (0.38)
3. Ability to actively engage with digital services	2.66 (0.70)	2.61 (0.72)	2.71 (0.60)
4. Feel safe and in control	2.83 (0.49)	2.78 (0.50)	2.95 (0.45)
5. Motivated to engage with digital services	2.59 (0.54)	2.64 (0.59)	2.67 (0.48)
6. Access to digital services that work	2.64 (0.46)	2.61 (0.45)	2.67 (0.43)
7. Digital services that suit individual needs	2.43 (0.58)	2.43 (0.57)	2.44 (0.54)

### Vignettes Developed

#### Cluster Analyses

Due to missing data, 198, 200 and 112 respondents were included for the cluster analyses of Site 1, Site 2, and Site 3, respectively. The results identified 8 (Site 1), 9 (Site 2), and 8 (Site 3) groups of respondents with different eHealth literacy profiles. The profiles from the cluster analyses were then combined with demographics, health conditions and technology use to provide a detail picture of the characteristics of each profile (see Table 4 for eHealth literacy profile of Site 1 and Supplementary Tables 1, and 2 for profiles of Site 2 and 3).

**Table 4:** Example of and eHealth literacy profiles based on an eight-cluster solution for Site 1 respondents.

**Cluster**	**A**	**B**	**C**	**D**	**E**	**F**	**G**	**H**
Number of respondents	6	24	39	51	43	17	17	1
% in sample	3.0	12.1	19.7	25.8	21.7	8.59	8.59	0.51
**eHLQ Mean score (SD)/ Score range: 1–4**								
1. Using technology to process health information	3.73 (0.24)	3.27 (0.33)	2.73 (0.31)	2.69 (0.40)	2.21 (0.25)	2.51 (0.30)	1.38 (0.32)	1.00
2. Understanding of health concepts and language	3.93 (0.16)	3.43 (0.34)	2.98 (0.22)	2.90 (0.28)	2.68 (0.28)	2.66 (0.40)	2.85 (0.37)	1.20
3. Ability to actively engage with digital services	3.77 (0.15)	3.40 (0.40)	3.11 (0.33)	2.67 (0.46)	2.33 (0.45)	2.57 (0.35)	1.36 (0.36)	1.00
4. Feel safe and in control	3.83 (0.23)	3.28 (0.47)	2.93 (0.24)	2.72 (0.32)	2.80 (0.23)	1.93 (0.31)	2.79 (0.38)	1.60
5. Motivated to engage with digital services	3.77 (0.23)	3.17 (0.34)	2.79 (0.23)	2.71 (0.25)	2.21 (0.30)	2.54 (0.44)	1.69 (0.40)	1.00
6. Access to digital services that work	3.56 (0.29)	3.08 (0.39)	2.90 (0.22)	2.72 (0.30)	2.35 (0.22)	2.20 (0.31)	2.11 (0.29)	1.17
7. Digital services that suit individual needs	3.46 (0.46)	2.96 (0.34)	2.98 (0.14)	2.40 (0.29)	2.09 (0.32)	2.02 (0.13)	1.41 (0.40)	1.00
**Sociodemographic characteristics**								
Mean age	52.7	44.3	50.0	51.4	57.0	49.3	71.3	93.0
% Female	83.3	62.5	61.5	54.9	58.1	58.8	76.5	100.0
% Do not speak english at home	16.7	25.0	25.6	31.4	34.9	47.1	64.7	100.0
Average education	5.6	5.0	5.1	5.0	4.6	5.4	2.8	2.0
Average IRSD10	7.2	5.9	6.4	5.2	5.8	6.3	3.9	3.0
% Have private health insurance	50.0	54.2	61.5	58.8	60.5	64.7	17.7	0.0
**Health conditions**								
% No long-standing health condition	50.0	62.5	56.4	47.1	39.5	58.8	47.1	0.0
% Arthritis	16.7	4.2	10.3	9.8	16.3	17.7	23.5	100.0
% Cancer	0.0	0.0	0.0	0.0	0.0	0.0	5.9	0.0
% CVD/heart disease	0.0	8.3	12.8	21.6	14.0	5.9	17.7	0.0
% Diabetes	16.7	0.0	7.7	11.8	11.6	5.9	11.6	0.0
% Respiratory condition	16.7	4.2	2.6	2.0	0.0	5.9	5.9	0.0
% Anxiety	16.7	20.8	7.7	13.7	16.3	23.5	5.9	0.0
% Depression	16.7	12.5	10.3	15.7	20.9	17.6	0.0	100.0
Average number of health conditions	1.2	0.7	0.6	1.0	1.0	0.8	0.8	2.0
Average self-perceived health status	2.0	2.4	2.6	2.8	2.9	2.7	3.0	3.0
**Technology use**								
% Use computer	100.0	83.3	89.7	68.6	69.8	94.1	17.7	0.0
% Use mobile phone/smartphone	100.0	95.8	94.9	94.1	86.0	100.0	52.9	0.0
% Use tablet	66.7	62.5	64.1	51.0	30.2	58.8	23.5	0.0
Average number of digital devices	2.7	2.5	2.5	2.1	1.9	2.6	0.9	0.0
% Use email	100.0	91.7	89.7	80.4	60.5	88.2	11.8	0.0
% Use text messaging	100.0	91.7	87.2	84.3	60.5	88.2	41.2	0.0
% Use facebook	83.3	79.2	64.1	60.8	46.5	41.2	17.7	0.0
Average number of digital platforms	4.7	3.0	3.6	3.0	2.2	2.9	0.9	0.0
% Looked for information online	100.0	91.7	89.7	80.4	62.8	94.1	17.7	0.0
% Monitored health digitally	66.7	62.5	48.7	23.5	18.6	52.9	0.0	0.0

*The eHLQ scores are highlighted using the traffic light system of color coding as recommended in the Ophelia process (56). Cells colored green represented higher scores, the range of yellow represent medium scores and red indicate lower scores. Education is represented by 6 categories: 1 = Did not complete primary school, 2 = Completed primary school, 3 = Did not complete secondary school, 4 = completed secondary school, 5 = Completed trade Certificate/Diploma/TAFE, 6 = Completed University or above. IRSD10 = The Index of Relative Socio-economic Disadvantage Decile 2016, ranking within Australia. This index is based on information provided by the Australian Bureau Statistics (58), postcodes are divided into 10 ranks with higher number indicating more advantaged suburbs. Self-perceived health status is represented by 5 categories: 1 = Excellent, 2 = Very good, 3 = Good, 4 = Fair, 5 = Poor*.

As shown in Table 4, among the 8 clusters of Site 1, 3 clusters (Clusters A to C) had generally higher scores across the 7 scales and 5 clusters (Clusters D to H) had lower scores but with different patterns. While the overall eHealth literacy scores of Site 1 demonstrated that respondents generally felt safe with digital health systems, the cluster analysis uncovered people from Cluster F who tend to report otherwise. Site 1 also had a “cluster” with only one member. The “cluster” was retained because older community members tended to refuse to participate in a survey about eHealth and this “cluster” provided some insights into a group of older people who were most likely being left behind in the digital age. For Site 2, the 9 clusters demonstrated very different patterns (see Supplementary Table 1). People from Cluster B with higher scores in scales relating to technology use had scores in “Scale 6 Access to digital services that work” and “Scale 7 Digital services that suit individual needs” comparable to the lower eHealth literacy clusters. There were also 2 clusters (Clusters D and F) reporting lower scores in “Scale 4 Feel safe and in control,” indicating lack of trust in eHealth systems for the people from these clusters. While the overall eHealth literacy scores of Site 3 in Scale 4 was higher than the other 2 sites, there were also 2 clusters (Clusters A and D) with lower scores in Scale 4, indicating concern for online security (see Supplementary Table 2). For the 2 clusters with the lowest scores in scales relating to technology use in Site 2 and Site 3, both clusters had scores in “Scale 2 Understanding of health concepts and language” that were comparable to the other higher eHealth literacy clusters, indicating strengths among these clusters in this eHealth literacy domain. See Table 5 for a summary description of the clusters.

**Table 5:** Description of clusters and vignettes.

**Cluster**	**Description**	**Vignette**
		**developed**
**Site 1**		
A	Tech-savvy and well-resourced	No
B	Young and digitally active	No
C	Good digital skills, healthy and digitally active	No
D	Average digital skills but digital active	Yes
E	Not interested in using technology but think eHealth is fine	Yes
F	Willing to use technology but not for health with concern about privacy	Yes
G	Good understanding of health with limited digital skills	Yes
H	No access or skills to use technology and limited understanding of health	No
**Site 2**		
A	Tech-savvy and well-resourced	No
B	Tech-savvy but poor access to useful digital services	No
C	Good digital skills and comfortable with eHealth	No
D	Good digital skills but concern about privacy	Yes
E	Limited digital skills but think eHealth maybe useful	Yes
F	Good digital skills but concerns about privacy and poor access to suitable digital services	Yes
G	Not interested in using technology but think eHealth is fine	Yes
H	Limited digital skills and not interested in technology	Yes
I	Good understanding of health and do not see technology useful	Yes
**Site 3**		
A	Tech-savvy, healthy and well-resourced	No
B	Tech-savvy with confidence in eHealth systems	No
C	Good digital skills and good understanding of health	No
D	Good digital skills but concern over privacy and poor experience with digital services	Yes
E	Average digital skills with limited access to suitable digital services	Yes
F	Average digital skills with poor access to suitable digital services	Yes
G	Limited digital skills but think eHealth is fine	Yes
H	Limited digital access and skills but good understanding of health	Yes

#### Vignettes

Five respondents, one male and four females aged from 53 to 75, were interviewed and provided further insights into people's experiences in using digital health. Based on the cluster analyses and interviews, 4, 6, and 5 vignettes were developed for clusters with different patterns of eHealth literacy strengths and weaknesses for Sites 1 to 3, respectively. A vignette for Cluster H of Site 1 was not generated because there was only one member. See Table 5 for descriptions of clusters with vignettes developed. See Table 6 for an example vignette and Supplementary Table 3 for all vignettes developed for the co-design workshops.

**Table 6:** Example of a vignette – Cluster G (Maria) of Site 1.

**Number of respondents**	**% in sample**	**Mean age**	**1. Using technology to process health information**	**2. Understanding of health concepts and language**	**3. Ability to actively engage with digital services**	**4. Feel safe and in control**	**5. Motivated to engage with digital services**	**6. Access to digital services that work**	**7. Digital services that suit individual needs**
17	9	71.3	1.38	2.85	1.36	2.79	1.69	2.11	1.41
Maria is a cheerful 82-year-old grandma with primary school education. She speaks limited English but can manage basic daily conversations. Living with her husband, she has two daughters and five grandchildren, who live close by. Having arthritis does not stop her from doing what she loves most – cooking for her family.									
Maria's daughter gave her a mobile phone last year and her grandson tried to teach her to use it without success. They ring her, but she never answers her phone either because she doesn't hear the phone ring, or she just keeps pressing the wrong buttons. The buttons are just too small, and she can hardly see them.									
Reading text messages is another next to impossible task. She has given up learning as she believes she will die soon, so, there is no need to learn these “new” digital technologies. She notices that her family doctor types her information into his computer, but she has no idea what that means. She knows you can find health information on the internet, but she strongly believes that you should always ask health professionals for advice, not the internet.									

### Digital Health Literacy-Related Improvement Activities Arising From Ideas Generation Workshops

Community member workshops included 6, 8, and 7 respondents at Sites 1 to 3, with 12 recruited from the cross-sectional survey who indicated interest and 9 from referrals by managers of the participating sites. Participants of the health professional workshops conducted at Sites 1 and 3 (4 and 5 participants, respectively), were recruited by senior management. The workshop at Site 2, attended by 26 health professionals, was undertaken as part of the monthly program meeting.

The personas embodied in the vignettes were well-recognized as persons or patients familiar to workshop respondents, prompting engaging discussion at all workshops. Some respondents even identified themselves as certain vignettes. The main problems identified by workshop respondents included lack of digital skills, lack of access to credible and reliable online health resources, concern about internet security, inadequate understanding of one's own health condition, using inappropriate digital devices and having eHealth systems that were difficult to use (see Supplementary Tables 4–6). The issues were similar across the 3 sites except Site 3 had the unique problem of no access to internet connection due to its regional location. Several respondents also pointed out that not using technology should not be regarded as a problem *(“She's got no problem.”* – Site 1 community member; “*Technology is not going to make him healthy.”* – Site 2 health professional).

At Site 1, 4 vignettes were presented which generated 32 solutions from the 2 workshops. Thematic analysis revealed 9 themes or strategies across the 32 ideas. Three of the strategies related to technology use such as providing access and support to skill training and reliable resources. Other strategies were more diverse such as ensuring effective communication with clients, harnessing social support, motivating clients to engage with own health, using multi-disciplinary approach to health care, capacity building for health professionals and ensuring access to both conventional and digital health services. A total of 17 ideas were targeted at the individual level, 11 at the policy level and 2 each for the practitioner and family levels. At Site 2, 6 vignettes were presented which generated 43 solutions across 10 themes where 15 were targeted at the individual, 17 ideas on the policy level, 5 on the practitioner level and 6 on the family or social level. At Site 3, 32 solutions were generated from 5 vignettes representing 10 strategies, where 24 were targeted at the individual, 6 at the policy level and 1 each for the practitioner and family levels. The themes at Sites 2 and 3 were the same as Site 1 except the two sites had an additional strategy about technology, which was to provide eHealth systems that meet different needs. See Table 7 for the thematic analysis results of strategies, number of solutions and some examples of solutions at the 3 sites.

**Table 7:** Co-designed strategies and number of solutions.

**Strategies**		**Number of solutions**		
		**Site 1**	**Site 2**	**Site 3**
1	Provide training or encourage use of technologies	7	4	5
	Examples of solutions:			
	• Advertise or provide access to technology training programs			
	• Provide a “digital navigator” to interact with clients in the waiting room to provide information or assist in using digital devices			
	• Support clients to choose appropriate digital device(s)			
2	Provide access to reliable and trustworthy eHealth resources	3	3	5
	Examples of solutions:			
	• Give clients specific links to navigate to appropriate websites •Sharing of consumer-focused eHealth resources between partner organizations			
	• Establish a way that the clinic's recommended digital services and apps can be easily downloaded by clients to their own devices			
3	Support clients with concerns on privacy and security of eHealth systems	3	5	3
	Examples of solutions:			
	• Educate clients on how eHealth services are provided with security and privacy considerations			
	• Advocate government to take responsibility in ensuring the safety and security of electronic health records			
	• Provide a health summary in physical form if client decides not to use electronic health records			
4	Provide technologies and eHealth systems that meet different needs	–	3	1
	Examples of solutions:			
	• Involve users when developing websites or digital technologies to match their needs and skills			
	• Advocate government to ensure electronic health records are up to date			
	• Ensure organization information technology systems are working smoothly to work with clients efficiently			
5	Ensure effective communication to meet individual needs	4	8	2
	Examples of solutions:			
	• Provide health information in multiple formats such as prints, audio, video, diagrams, large print or appropriate languages			
	• Encourage clinicians to use plain language and write down information and instructions for clients			
	• Support practitioners with access to culturally appropriate resources			
6	Harness family and social support	4	9	1
	Examples of solutions:			
	• Encourage volunteers, friends or family members to provide regular practice in using technologies through one-on-one coaching or mentoring			
	• Encourage and support family members to manage health for the elderly			
	• Provide a space and opportunities for social networking among clients to share good health information			
7	Motivate clients to actively engage with own health	6	3	6
	Examples of solutions:			
	• Educate clients about their health conditions, assist them to set up personal goals and link their interest to health-promoting activities			
	• Connect clients' interest to technologies and provide positive experiences such as using iPad to demonstrate exercise or provide feedback during consultations			
	• Provide access to community educators or nurses to promote understanding of own health condition			
8	Use a tailored and multi-disciplinary approach to health care	2	2	5
	Examples of solutions:			
	• Refer clients to key services, e.g., mental health, exercise, etc.			
	• Support clinicians with better access to medical history of clients (with clients' consent) to facilitate a team-approach to health care			
	• Provide a comprehensive multi-disciplinary “one-stop-shop” in one session with content that really helps clients			
9	Build capacity for evidence-based practice	1	2	1
	Examples of solutions:			
	• Ensure health professionals have a genuine understanding of available health education courses			
	• Provide clinicians with ongoing professional development on eHealth			
	• Explore best practice and health evidence and support clinicians with ongoing professional development			
10	Provide access to conventional and digital health services	2	4	3
	Examples of solutions:			
	• Connect with clients using appropriately tailored communication platform			
	• Provide clients with summaries of medical history and/or medication in printed formats			
	• Keep in mind that there are people who are “out of the web” in strategic planning			

While the strategies were generally consistent across the 3 sites, some of the solutions could be very similar but some were unique to a certain site. Common solutions around technology use included providing access to technology training programs, support clients to choose appropriate digital devices, and give links to reliable and trustworthy websites. Another common solution recommended was to provide physical handout or different formats of health information, demonstrating that eHealth literacy needs could be met by both technological or non-technological solutions. In addition, the solutions might focus on the skills of individual clients but could also target health professionals such as ensuring clinicians had adequate resources to support clients. Advocating the government in terms of electronic health records safety was another common solution suggested to address people's concern over privacy and security. However, the practice of each organization could also lead to some unique solutions. For example, for the strategy of “Ensure effective communication to meet individual needs,” Site 1 had the idea of “Ensure the data collected at online booking are available at the patients” appointments' as online booking was available in this site. For Site 2, this strategy included the idea of “Ensure interpreters are available for culturally and linguistic diverse communities” as this site had a culturally diverse client base. Details of the solutions are presented in Supplementary Tables 4–6.

### Evidence for the Application of the Digital Ophelia Process

#### Rating Questionnaire

The intervention ideas rating questionnaire was completed by 4, 3, and 3 executives or staff members at Sites 1, 2, and 3, respectively. About half of the ideas were rated very important or essential and many ideas were rated feasible by individual respondents at all sites. There were no clear unimportant solutions. There were also a range of opinions on estimated current practice. See Supplementary Tables 4–6 for the very important or essential ideas rated by all respondents at the 3 sites.

#### Proof-of-Concept Application

In mapping the results of the study against the 8 guiding principles of the Ophelia process (Table 1), many of the principles were mostly or partially applied while further evidence was required for the sustainable principle (P6) as this study only involved Phase 1 of the process. One of the aims of this study was to specifically develop intervention ideas for health improvement (P1 Outcome focused). However, sites generally viewed the project as a pilot project and did not see it as their own initiative for system and services improvement, leading to partial application of this principle. By using the eHLQ for eHealth literacy needs assessment, the vignettes provided insights into the eHealth literacy challenges of different client groups and helped identify local needs (P4 Needs-diagnostic approach). The participation of community members and health professionals at co-design workshops ensured that relevant stakeholders were engaged (P3 Co-design approach), providing local wisdom (P5 Driven by local wisdom) to help generate responsive actions addressing the different needs of clients (P7 Responsiveness). For the needs assessment using the eHLQ, respondents were provided with the option of face-to-face interviews to ensure that older people and people with lower literacy were included as an equity driven strategy. The cluster analyses further ensured that small groups facing eHealth literacy challenges were included, leading to intervention ideas such as non-digital or culturally appropriate health information to meet the diverse needs of different client groups. Hence, P2 Equity driven was mostly applied. The solutions co-designed at the workshops spanned across 4 levels including the individual, family, practitioner and policy (P8 Systematically applied) but feasibility of the implementation of the ideas needed to be established. Besides, evidence for the sustainability (P6) of the ideas required further application of the remaining Ophelia phases. A summary of the evidence on how the 8 guiding Ophelia principles have been operationalized are presented in Supplementary Table 7.

## Discussion

By using the Ophelia process with the eHLQ as the needs assessment tool, numerous solutions to improve health and equity outcomes were generated in 3 disparate health settings. While similar strategies were identified across the settings, solutions that addressed the specific needs of subgroups and were fit for the local context were suggested by clients and health professionals through co-design. About half of the ideas were rated very important or essential by the relevant executives or staff of the participating sites and the proof-of-concept also showed that most of the guiding principles of the Ophelia process were applied. As such, a clear and reproducible pathway to co-designing solutions in the digital context has been demonstrated in this study. The results also demonstrate that the Ophelia process can be adapted into the digital context and applied to co-design solutions addressing eHealth literacy needs.

A feature of the Ophelia process is the use of vignettes to provide real-life stories of people facing challenges in using digital health. The vignettes were derived from the eHLQ, a multi-dimensional tool assessing the 7 domains of eHealth literacy. As such, the experience was not restricted to digital skills or evaluating online health information; motivation, privacy concern, interaction of digital health systems or understanding of health concepts were also assessed. Understanding the various aspects of eHealth literacy will have implications for the planning of solutions to address eHealth literacy weaknesses. For example, addressing privacy concerns instead of simple digital training will likely be more useful to motivate people should internet security stand in the way of using technology. On the other hand, people with limited digital skills but who believe that digital health is useful will likely benefit from some skills training.

A strength of the Ophelia process is the use of cluster analysis to gain in-depth understanding of the needs of subgroups. The pattern of the total scores of each organization was similar (Table 3). If these scores and descriptions are to be used to “tailor” digital health solutions, these organizations may end up providing a limited range of similar solutions for their clients. By using cluster analysis, 8 to 9 clusters with different patterns of eHealth literacy strengths and weaknesses in each setting were identified. These clusters provided valuable additional information and insights into the different needs of various client groups. While the overall mean scores indicated that clients were generally comfortable with the security of eHealth services, there were clusters within all 3 organizations that expressed privacy concerns, especially in Site 2 where 19% of the sample (clusters D and F) had doubts over how their health data were used (Supplementary Table 1). Hence, in using traditional analysis such as mean scores and descriptions to “tailor” solutions, the privacy concerns of 19% of Site 2 clients will likely be overlooked. If a widescale digital solution was implemented in Site 2 based on the total scores, about on fifth of the clients may hesitate to adopt the new service. The cluster analyses also revealed certain groups of people who were likely to be facing challenges in the digital age, such as the single-member cluster of Site 1 (Cluster H), a “cluster” that represented older community members who were most likely to have no skills or access to digital technologies. Instead of ignoring these small clusters as recommended in traditional cluster analysis approaches (46, 47), the retention of such clusters as guided by the Ophelia process will be in a better position to promote equity. To bridge the digital divide, it is essential to identify people who are experiencing challenges in accessing and using digital health information and services. As such, their needs are considered when developing and implementing quality improvements and interventions.

The Ophelia approach to develop vignettes also includes examining demographics and personal experiences. The resulting mix of information allows for a lively description of eHealth literacy strengths and weaknesses along with potential factors that may impact eHealth literacy, with the expectation that different solutions may be generated for different vignettes. For example, both Site 1 and Site 2 have a culturally diverse client base and strategies to address language barriers are likely to be needed, while these strategies may not be essential to Site 3 where clients are predominately English-speaking or can communicate in English as they choose to attend a private medical practice. The inclusion of personal experiences derived from the semi-structured interviews also allows users to put a voice into the vignettes and adds valuable insights into the eHealth literacy profiles. For example, the lack of internet infrastructure in the regional area revealed by an interviewee provides clues into one of the reasons for limited access to digital health services. Hence, strategies to meet needs such as the lack of internet infrastructure for regional clients will be required but not for metropolitan users.

The use of vignettes to describe people's needs and challenges in the Ophelia process follows recommendations that vignettes should be used to assist digital health development (58–60). This approach, as in the Ophelia process, generates a safe and pragmatic environment where respondents genuinely engage with familiar and concrete material in text, oral and narrative formats. Respondents then draw directly on their personal practices and experiences to accumulate a wide range of thoughtful and realistic solutions to the multidimensional lives embodied in the vignettes. Respondents frequently recounted their own success stories of helping people facing similar challenges. Community member workshops generate a different mix of solutions with different emphasis when compared with the professionals. In this way, across multiple workshops and with inputs from many different community members and types of professionals, a whole of system solution is incrementally generated. The use of the eHLQ clearly generated diverse and meaningful vignettes that harness the different perspectives and wisdom of community members and health professionals in the co-design process.

The variety of solutions to tackle eHealth literacy needs generated through the co-design workshops is in stark contrast to the current eHealth literacy interventions found in the literature that usually focus only on building digital skills (30, 61). The results also feature ideas targeted at different levels, including family, practitioners and organization policies, providing a holistic approach to solutions instead of placing the burden of change solely on the individuals. A main finding from the co-design workshops is the recommendation of the use of not only digital health, but also conventional health solutions based on eHealth literacy needs. The ideas were in response to the needs of people who might not have access or skills to use technology for health. In fact, many workshop respondents indicated that not using technology should not be regarded as a problem and the strategy of “Providing access to conventional and digital health services” is a consistent theme for all 3 organizations. Thus, the co-design process revealed that a non-digital solution can also be a way to bridge the digital divide. Health organizations need to recognize that technology is only a means, not an end (62). Equitable access should not just refer to access to the same resources. The resources should be flexible enough to suit individual needs as people may be facing different challenges and they should be allowed to lead lives of their own preferences (63). Thus, the Ophelia process has provided an equity driven and responsive approach to co-design solutions in the digital context, without being confined to only digital solutions. This result also resonates with the digital health recommendations of the WHO which stipulates that provision of non-digital services should not be excluded when access, acceptability or affordability of technologies are in question for target communities (12).

To make progress in developing digital health solutions, it is recommended that the Ophelia process be used in the digital context by beginning with needs assessment using the eHLQ or in combination with the HLQ. Armed with an understanding of users' eHealth literacy and health literacy needs, developers can then co-design and implement initiatives through participation to ensure user' needs are addressed. As health services continue to become digitalized, it is high time for health care organizations and policymakers to mandate the use of a co-design and equity driven approach, such as the Ophelia process, to engage with users and ensure digital health systems are adopted to realize the potential of health improvement.

### Limitations

A limitation of this study in operationalizing the Ophelia principles is the participating sites viewing the study as a pilot/proof-of-concept study applying only the first of the three Ophelia phases rather than an organizationally-owned and led process to produce service and system improvements for their organizations (3 Ophelia phases). As an underlying aim of the Ophelia co-design approach (25, 26), this reduced sense of ownership may have led to low participation of workshop respondents at the 2 medical practices. Nevertheless, the limited number of workshop respondents at the 2 primary care clinics still generated 32 solutions, shedding light on some important and useful ideas for the clinics. On the other hand, the commitment of senior management in assisting recruitment of respondents throughout the study demonstrated the importance of strong organizational leadership in the implementation of a co-design process. In a recent systematic review of the implementation of care delivery technologies for older adults, organizational leadership was identified as one of the key influencing factors (64). To ensure any co-design program, such as the Ophelia process, can be successfully implemented, strong organizational leadership to create and foster a culture of partnership and engagement among the workplace is essential (65, 66).

It should also be noted that the 3 organizations were not highly digitally active at the time of the study. Their websites were generally simple and straightforward. Only Site 1 offered online appointments and Site 3 had telehealth services while only Site 1 and Site 2 were on Facebook. Further work needs to be done to explore the Ophelia process in digitally active health settings, such as organizations which are active on social media and offer mainly online resources as well as interactive activities. Another possible limitation was the absence of workshop respondents with expertise in information technology who may offer a professional technological perspective to the eHealth literacy needs discussed.

Finally, the ideas were not implemented and evaluated due to the scope of the study. While many of the solutions were suggested based on personal success experiences and the ideas also received the support of expert validation, whether these ideas can assist improvement of health and equity outcomes has yet to be tested. With only Phase 1 of the Ophelia process being undertaken, implementation of Phases 2 and 3 of the Ophelia process for the co-production, implementation and evaluation of interventions is needed to provide further evidence of the feasibility of the process in the digital context.

### Conclusion

By harnessing collective creativity, the Ophelia process has been shown to efficiently engage stakeholders in the co-design of digital and other solutions with the potential to improve health and equity outcomes. The co-design process generated diverse solutions targeting individuals as well as family, medical practitioners and organization policies. Of importance is the inclusion of non-digital solutions as one of the potential ways to bridge the digital divide when most current solutions focus only on digital skills. It serves as a timely reminder that health organizations and policymakers must acknowledge and be responsive to the different challenges faced by diverse people to ensure that the digital gap is addressed. Strong organizational leadership is also needed to create a culture of partnership to ensure the success of a co-design process. As such, taking a co-design approach to the development of digital health initiative will ensure that it is not another step toward the widening of health inequalities but a step closer to health equity.

## Data Availability Statement

The original contributions presented in the study are included in the article/Supplementary Materials, further inquiries can be directed to the corresponding author/s.

## Ethics Statement

The studies involving human participants were reviewed and approved by Deakin University Human Research Ethics Committee (HEAG-H 146_2017). Returning of the survey questionnaire was considered implied consent. Oral consent was obtained from participants of semi-structured interviews. Participants of workshops provided their written informed consent to participate in the workshops.

## Author Contributions

CC led the data collection, undertook the data analysis, and reviewed by other authors. CC drafted the manuscript. All authors reviewed and provided feedback to all manuscript iterations, approved the final manuscript, and contributed to the conceptualization of the study, methods, and analysis.

## Conflict of Interest

The authors declare that the research was conducted in the absence of any commercial or financial relationships that could be construed as a potential conflict of interest.
