# Supplementary material for: Co-designing eHealth and Equity Solutions: Application of the Ophelia (Optimizing Health Literacy and Access) Process
Source: Front Public Health. 2020 Nov 20;8:604401. doi: 10.3389/fpubh.2020.604401 (PMC7718029; doi:10.3389/fpubh.2020.604401)
Supplement: Supplementary file 2 [file Table_2.DOCX]

**Supplementary Material 2 | The eight eHealth literacy profiles of Site 3 respondents**

| **Cluster** | **A** | **B** | **C** | **D** | **E** | **F** | **G** | **H** |
| --- | --- | --- | --- | --- | --- | --- | --- | --- |
| Number of respondents | 3 | 16 | 25 | 6 | 35 | 13 | 10 | 4 |
| % in sample | 2.7 | 14.3 | 22.3 | 5.4 | 31.3 | 11.6 | 8.9 | 3.6 |
| **eHLQ Mean score (SD)/ Score range: 1 – 4** | | | | | | | | |
| 1. Using technology to process health information | 3.67  (0.31) | 3.14  (0.36) | 2.92  (0.26) | 2.73  (0.21) | 2.48  (0.28) | 2.52  (0.17) | 2.25  (0.22) | 1.25  (0.19) |
| 2. Understanding of health concepts and language | 3.87  (0.12) | 3.31  (0.37) | 3.00  (0.15) | 3.17  (0.20) | 2.82  (0.23) | 2.85  (0.21) | 2.26  (0.23) | 2.75  (0.34) |
| 3. Ability to actively engage with digital services | 3.93  (0.12) | 3.05  (0.42) | 3.04  (0.29) | 3.48  (0.29) | 2.55  (0.39) | 2.46  (0.38) | 2.09  (0.24) | 1.20  (0.28) |
| 4. Feel safe and in control | 3.33  (0.50) | 3.61  (0.34) | 2.98  (0.18) | 2.27  (0.10) | 3.03  (0.22) | 2.48  (0.35) | 2.75  (0.25) | 2.45  (0.72) |
| 5. Motivated to engage with digital services | 3.80  (0.20) | 3.01  (0.28) | 3.05  (0.24) | 2.73  (0.27) | 2.49  (0.27) | 2.49  (0.29) | 2.06  (0.16) | 1.70  (0.62) |
| 6. Access to digital services that work | 3.44  (0.25) | 3.05  (0.31) | 2.89  (0.26) | 2.41  (0.35) | 2.66  (0.26) | 2.19  (0.37) | 2.23  (0.25) | 2.17  (0.53) |
| 7. Digital services that suit individual needs | 3.50  (0.50) | 2.78  (0.39) | 2.95  (0.10) | 2.14  (0.39) | 2.32  (0.28) | 2.08  (0.26) | 1.93  (0.31) | 1.00  (0.00) |
| **Sociodemographic characteristics** | | | | | | | | |
| Mean age | 33.0 | 54.8 | 57.2 | 52.0 | 56.9 | 47.0 | 59.2 | 56.3 |
| % Female | 100.0 | 56.3 | 56.0 | 16.7 | 74.3 | 69.2 | 80.0 | 0.0 |
| % Do not speak English at home | 0.0 | 18.8 | 4.0 | 0.0 | 5.7 | 7.7 | 10.0 | 0.0 |
| Average education | 4.7 | 4.8 | 4.9 | 5.3 | 4.3 | 5.2 | 4.6 | 4.5 |
| Average IRSD10 | 9.0 | 7.4 | 6.0 | 7.3 | 6.0 | 6.2 | 5.3 | 6.5 |
| % Have private health insurance | 100.0 | 43.8 | 40.0 | 83.3 | 54.3 | 69.2 | 30.0 | 25.0 |
| **Health conditions** | | | | | | | | |
| % No long-standing health condition | 66.7 | 31.3 | 64.0 | 50.0 | 40.0 | 38.5 | 30.0 | 25.0 |
| % Arthritis | 0.0 | 18.8 | 24.0 | 0.0 | 25.7 | 46.2 | 40.0 | 25.0 |
| % Cancer | 0.0 | 0.0 | 0.0 | 0.0 | 5.7 | 0.0 | 0.0 | 0.0 |
| % CVD/heart disease | 0.0 | 31.3 | 8.0 | 0.0 | 14.3 | 30.8 | 0.0 | 25.0 |
| **Cluster** | **A** | **B** | **C** | **D** | **E** | **F** | **G** | **H** |
| % Diabetes | 0.0 | 12.5 | 4.0 | 16.7 | 5.7 | 7.7 | 20.0 | 0.0 |
| % Respiratory condition | 0.0 | 12.5 | 8.0 | 16.7 | 17.1 | 15.4 | 10.0 | 0.0 |
| % Anxiety | 0.0 | 18.8 | 8.0 | 0.0 | 29.0 | 23.1 | 10.0 | 25.0 |
| % Depression | 0.0 | 12.5 | 4.0 | 0.0 | 14.3 | 7.7 | 20.0 | 25.0 |
| Average number of health conditions | 0.3 | 1.4 | 0.6 | 0.7 | 1.1 | 1.5 | 1.3 | 1.0 |
| Average self-perceived health status | 1.3 | 3.0 | 2.5 | 3.0 | 2.8 | 3.2 | 3.4 | 3.3 |
| **Technology use** | | | | | | | | |
| % Use computer | 100.0 | 81.3 | 92.0 | 83.3 | 80.0 | 92.3 | 50.0 | 0.0 |
| % Use mobile phone/Smartphone | 100.0 | 100.0 | 88.0 | 83.3 | 100.0 | 100.0 | 90.0 | 50.0 |
| % Use tablet | 100.0 | 75.0 | 52.0 | 66.7 | 40.0 | 53.9 | 40.0 | 0.0 |
| Average number of digital devices | 3.0 | 2.6 | 2.3 | 2.3 | 2.2 | 2.5 | 1.8 | 0.5 |
| % Use email | 100.0 | 100.0 | 88.0 | 100.0 | 85.7 | 92.3 | 80.0 | 0.0 |
| % Use text messages | 100.0 | 100.0 | 88.0 | 83.3 | 91.4 | 84.6 | 70.0 | 50.0 |
| % Use Facebook | 100.0 | 62.5 | 68.0 | 50.0 | 57.1 | 76.9 | 50.0 | 0.0 |
| Average number of digital platforms | 6.0 | 3.0 | 3.2 | 2.3 | 2.8 | 3.4 | 2.3 | 0.5 |
| % Looked for information online | 100.0 | 100.0 | 84.0 | 83.3 | 80.0 | 92.3 | 70.0 | 25.0 |
| % Monitored health digitally | 100.0 | 68.8 | 56.0 | 50.0 | 22.9 | 23.1 | 10.0 | 0.0 |
| Notes: The eHealth Literacy Questionnaire scores are highlighted using the traffic light system of color coding as recommended in the Ophelia process (56). Cells colored green represented higher scores, the range of yellow represent medium scores and red indicate lower scores. Education is represented by 6 categories: 1 = Did not complete primary school, 2 = Completed primary school, 3 = Did not complete secondary school, 4 = completed secondary school, 5 = Completed trade Certificate/Diploma/TAFE, 6 = Completed University or above. IRSD10 = The Index of Relative Socio-economic Disadvantage Decile 2016, ranking within Australia. This index is based on information provided by the Australian Bureau Statistics (59), postcodes are divided into 10 ranks with higher number indicating more advantaged suburbs. Self-perceived health status is represented by 5 categories: 1 = Excellent, 2 = Very good, 3 = Good, 4 = Fair, 5 = Poor. | | | | | | | | |
