# Supplementary material for: Co-designing eHealth and Equity Solutions: Application of the Ophelia (Optimizing Health Literacy and Access) Process
Source: Front Public Health. 2020 Nov 20;8:604401. doi: 10.3389/fpubh.2020.604401 (PMC7718029; doi:10.3389/fpubh.2020.604401)
Supplement: Supplementary file 3 [file Table_3.DOCX]

**Supplementary Materials 3 | Vignettes developed for co-design workshops**

**Site 1 Cluster D (Jennifer) – Average digital skills but digitally active**

| Number of respondents | % in sample | Mean age | 1. Using technology to process health information | 2. Understanding of  health concepts and language | 3. Ability to actively  engage with digital services | 4. Feel safe and  in control | 5. Motivated to engage with digital services | 6. Access to digital  services that work | 7. Digital services  that suit individual needs |
| --- | --- | --- | --- | --- | --- | --- | --- | --- | --- |
| 51 | 26 | 51.4 | 2.69 | 2.90 | 2.67 | 2.72 | 2.71 | 2.72 | 2.40 |

Jennifer is a full-time mother, in her mid-forties, with two sons. She worked as an administrative assistant after completing secondary school but stopped working after she gave birth to her second son who is now 3 years old. Her older son is 6 years old. She had gestational diabetes during her second pregnancy, but this seems to have gone away.

Her doctor suggests her to maintain a healthy lifestyle as she is still a little overweight. She wants to keep track of her body weight but finds this difficult. She has a laptop, a smartphone and an iPad and is active on Facebook and Instagram where she regularly posts her sons’ latest photos and connects with her friends.

She uses the internet to find health information, however, such information may not always help her understand health problems as some of them are giving conflicting information. She also feels that most of these websites do not make it easy for her to get the information she needs. On the other hand, she does find it very convenient that she can access her family doctor online and make medical appointments anytime anywhere.

**Site 1 Cluster E (Michael) – Not interested in using technology but think eHealth is fine**

| Number of respondents | % in sample | Mean age | 1. Using technology to process health information | 2. Understanding of  health concepts and language | 3. Ability to actively  engage with digital services | 4. Feel safe and  in control | 5. Motivated to engage with digital services | 6. Access to digital  services that work | 7. Digital services  that suit individual needs |
| --- | --- | --- | --- | --- | --- | --- | --- | --- | --- |

| 43 | 22 | 57.0 | 2.21 | 2.68 | 2.33 | 2.80 | 2.21 | 2.35 | 2.09 |
| --- | --- | --- | --- | --- | --- | --- | --- | --- | --- |

Michael, aged 63, is a part-time plumber with high cholesterol but generally thinks his health is good. He takes his medication regularly and believes he understands his health condition reasonably well.

He is not keen on using digital technologies but has a mobile phone for easy communication with his clients. He attempted to use the internet to find health information when his wife was diagnosed with breast cancer. However, not only was the information difficult to read, it was also confusing, making it even harder to make decisions for his wife’s treatment. Adding to his confusion is that these health websites always change their user interface and he must learn his way around again and again.

He knows that his family doctor is uploading his and his wife’s health information onto the My Health Record* and he is happy about it. He believes digital health records can improve their healthcare. However, he’s never been onto the My Health Record website as he is not confident that he can enter data correctly and worries that he may mess up his data.

*My Health Record – a personal electronic health record in Australia

**Site 1 Cluster F (Anthony) – Willing to use technology but not for health with concern about privacy**

| Number of respondents | % in sample | Mean age | 1. Using technology to process health information | 2. Understanding of  health concepts and language | 3. Ability to actively  engage with digital services | 4. Feel safe and  in control | 5. Motivated to engage with digital services | 6. Access to digital  services that work | 7. Digital services  that suit individual needs |
| --- | --- | --- | --- | --- | --- | --- | --- | --- | --- |

| 17 | 9 | 49.3 | 2.51 | 2.66 | 2.57 | 1.93 | 2.54 | 2.20 | 2.02 |
| --- | --- | --- | --- | --- | --- | --- | --- | --- | --- |

Born in Lebanon, Anthony came to Melbourne 15 years ago with his wife to join his older brothers. He has an Accounting degree and speaks fluent English but trying to have his degree and experience recognized in Australia has not been easy. He eventually found a full-time job in a busy accounting firm, but he constantly worries about his performance on the job.

Anthony is generally in good health except for the stress from work. To combat anxiety, he goes to the gym at least three times a week. He has a computer, smartphone and a tablet but he doesn’t use these devices for health reasons. He doesn’t feel safe to organize his personal data and health information digitally as his computer has been hacked. While he uses email, text message and Skype to connect with his friends and family, he does not have any social media account. He has big concerns over privacy, citing the misuse of personal data at Facebook. He does not believe his health data can be stored safely, as digital systems, like his personal computer, can always be hacked.

**Site 1 Cluster G (Maria) – Good understanding of health with limited digital skills**

| Number of respondents | % in sample | Mean age | 1. Using technology to process health information | 2. Understanding of  health concepts and language | 3. Ability to actively  engage with digital services | 4. Feel safe and  in control | 5. Motivated to engage with digital services | 6. Access to digital  services that work | 7. Digital services  that suit individual needs |
| --- | --- | --- | --- | --- | --- | --- | --- | --- | --- |

| 17 | 9 | 71.3 | 1.38 | 2.85 | 1.36 | 2.79 | 1.69 | 2.11 | 1.41 |
| --- | --- | --- | --- | --- | --- | --- | --- | --- | --- |

Maria is a cheerful 82-year-old grandma with primary school education. She speaks limited English but can manage basic daily conversations. Living with her husband, she has two daughters and five grandchildren, who live close by. Having arthritis does not stop her from doing what she loves most – cooking for her family.

Maria’s daughter gave her a mobile phone last year and her grandson tried to teach her to use it without success. They ring her, but she never answers her phone either because she doesn’t hear the phone ring, or she just keeps pressing the wrong buttons. The buttons are just too small, and she can hardly see them. Reading text messages is another next to impossible task. She has given up learning as she believes she will die soon, so, there is no need to learn these ‘new’ digital technologies. She notices that her family doctor types her information into his computer, but she has no idea what that means. She knows you can find health information on the internet, but she strongly believes that you should always ask health professionals for advice, not the internet.

**Site 2 Cluster D (David) – Good digital skills but concern about privacy**

| Number of respondents | % in sample | Mean age | 1. Using technology to process health information | 2. Understanding of  health concepts and language | 3. Ability to actively  engage with digital services | 4. Feel safe and  in control | 5. Motivated to engage with digital services | 6. Access to digital  services that work | 7. Digital services  that suit individual needs |
| --- | --- | --- | --- | --- | --- | --- | --- | --- | --- |

| 16 | 8.0 | 55.4 | 2.91 | 2.93 | 2.93 | 2.25 | 2.85 | 2.72 | 2.77 |
| --- | --- | --- | --- | --- | --- | --- | --- | --- | --- |

At the age of 58, David is the property manager of a local real estate agency. On top of his ongoing knee problems, he was diagnosed with high blood pressure two years ago, and the medication isn’t quite working as well as he hoped.

David takes his medication and monitors his blood pressure regularly. He is also seeing the Dietitian at the local community health service. He has become comfortable using the internet to find out more about his conditions. David also downloaded several fitness apps but deleted those that tracked his location. He uses the prescription app of his local pharmacy to help him refill his medication and books his medical appointments with his family doctor online. However, news of how Facebook had misused users’ data confirmed his suspicions regarding the safety of digital health records. He realizes that he doesn’t really have any idea how his data are being used and whether they are safe.

**Site 2 Cluster E (Barbara) – Limited digital skills but think eHealth maybe useful**

| Number of respondents | % in sample | Mean age | 1. Using technology to process health information | 2. Understanding of  health concepts and language | 3. Ability to actively  engage with digital services | 4. Feel safe and  in control | 5. Motivated to engage with digital services | 6. Access to digital  services that work | 7. Digital services  that suit individual needs |
| --- | --- | --- | --- | --- | --- | --- | --- | --- | --- |

| 14 | 7.0 | 63.6 | 2.54 | 2.47 | 2.25 | 2.70 | 2.85 | 2.80 | 2.39 |
| --- | --- | --- | --- | --- | --- | --- | --- | --- | --- |

Barbara, 64, works as a part-time retail assistant and is experiencing depression since her husband Robert passed away two years ago. She feels that life is no longer interesting without Robert. She has two adult children and five grandchildren who live not too far away.

Barbara is not really into technology but would like to be. She enjoys playing games on her iPad which is a gift from her son. Her granddaughter shows her how to use the internet to find knitting patterns, but she always forgets how to do it. However, she feels that if someone has more time to teach her, she should be able to use it better. She finds online health information difficult to understand, she believes one should check with the doctor and she prefers to just follow her doctor’s recommendations. Apparently, her family doctor has uploaded her information onto the My Health Record and she doesn’t see any problem with it. However, she is unsure what it is for and finds the website confusing.

**Site 2 Cluster F (Ming) – Good digital skills but concerns about privacy and poor access to suitable digital services**

| Number of respondents | % in sample | Mean age | 1. Using technology to process health information | 2. Understanding of  health concepts and language | 3. Ability to actively  engage with digital services | 4. Feel safe and  in control | 5. Motivated to engage with digital services | 6. Access to digital  services that work | 7. Digital services  that suit individual needs |
| --- | --- | --- | --- | --- | --- | --- | --- | --- | --- |

| 22 | 11.0 | 55.2 | 2.73 | 2.94 | 2.80 | 2.13 | 2.61 | 2.19 | 2.09 |
| --- | --- | --- | --- | --- | --- | --- | --- | --- | --- |

Ming, with a bachelor's degree in Industrial Engineering from the Nanjing University, is always into the latest gadgets. He moved to Melbourne from China about ten years ago with his wife and son when he was 45. He doesn’t have any immediate family here but has no problem meeting new friends through WeChat.

Ming is really quite healthy for his age but was recently diagnosed with high cholesterol and he is a little overweight. His doctor, also from China, has warned him of the risk of developing heart disease. He has since done some searching on the internet and is taking some Chinese herbs as recommended on some of the Chinese health websites. He seldom uses Australian health websites as his English is limited. While some of the sites have Chinese language option, he finds most of the translation difficult to understand. When he reads about the My Health Record on the local Chinese newspaper, he is feeling very concerned as he never trusts the government about privacy. He is just not sure who is using his records or whether they are being used appropriately.

**Site 2 Cluster G (James) – Not interested in using technology but think eHealth is fine**

| Number of respondents | % in sample | Mean age | 1. Using technology to process health information | 2. Understanding of  health concepts and language | 3. Ability to actively  engage with digital services | 4. Feel safe and  in control | 5. Motivated to engage with digital services | 6. Access to digital  services that work | 7. Digital services  that suit individual needs |
| --- | --- | --- | --- | --- | --- | --- | --- | --- | --- |

| 42 | 21.0 | 70.2 | 2.14 | 2.97 | 2.23 | 2.92 | 2.28 | 2.53 | 2.18 |
| --- | --- | --- | --- | --- | --- | --- | --- | --- | --- |

James, 71 years old and a former plumber, was enjoying semi-retirement until he needed heart bypass surgery last year. His wife is making sure that he has a healthy diet and he has stopped excessive drinking since the surgery. Since his doctor referred him to the heart health program at the local community health service, James has developed a better understanding of how to stay healthy.

His daughter bought him an iPhone, which he used a few times for step counts. He doesn’t think the function is useful. She also sets him up on Facebook, but he only looks at it occasionally, when helped, to see what’s happening with the family. The text size is too small, and he can only look at the pictures. For James, although he could have access to new technologies, it just doesn’t seem worthwhile to him. He would rather go fishing. If James needs health information, he would ask his doctors who he feels understand him well. James sees the value of having his family doctor and specialists sharing his medical information electronically – to him it seems like a safe and sensible thing to do.

**Site 2 Cluster H (Anna) – Limited digital skills and not interested in technology**

| Number of respondents | % in sample | Mean age | 1. Using technology to process health information | 2. Understanding of  health concepts and language | 3. Ability to actively  engage with digital services | 4. Feel safe and  in control | 5. Motivated to engage with digital services | 6. Access to digital  services that work | 7. Digital services  that suit individual needs |
| --- | --- | --- | --- | --- | --- | --- | --- | --- | --- |

| 18 | 9.0 | 59.9 | 2.04 | 2.29 | 2.12 | 2.38 | 2.00 | 2.12 | 2.00 |
| --- | --- | --- | --- | --- | --- | --- | --- | --- | --- |

Anna and her husband George came to Australia twenty years ago and have two daughters who have grown up and left home. At 62, she works as a cashier at a Greek grocery store. Being close to the Greek community, she doesn’t have to speak English a lot but can still manage daily English conversation.

Anna has been living with arthritis for some time and recently had a sprained ankle which led her to see a physiotherapist. The physiotherapist gave her some suggestions on exercise, but she never seemed to remember what to do afterwards. Her daughter bought her a mobile phone which she sometimes uses for calling. She is not using it for texting because she finds typing on the phone too difficult. While her girls want to get her a smartphone, she doesn’t think she can use it. In fact, she considers too much technology is bad for you. Anna feels her doctor will use her health data appropriately but has no idea how such data are being stored.

**Site 2 Cluster I (Doris) – Good understanding of health and do not see technology useful**

| Number of respondents | % in sample | Mean age | 1. Using technology to process health information | 2. Understanding of  health concepts and language | 3. Ability to actively  engage with digital services | 4. Feel safe and  in control | 5. Motivated to engage with digital services | 6. Access to digital  services that work | 7. Digital services  that suit individual needs |
| --- | --- | --- | --- | --- | --- | --- | --- | --- | --- |

| 16 | 8.0 | 73.6 | 1.34 | 2.99 | 1.25 | 2.69 | 1.70 | 2.17 | 1.50 |
| --- | --- | --- | --- | --- | --- | --- | --- | --- | --- |

Doris has been a housewife since she was married at the age of 20. Now 84, she has lung disease but can use her puffer without much problem. She also joins the Good Life Club at Carrington Health to make sure she maintains a good level of physical activity for her condition.

Doris has rarely looked at a computer or tablet and thinks it is beyond her. This doesn’t bother her as she doesn’t think technology can be used to improve her health. She trusts her doctor and her motto is ‘you should always go to your doctor’, not to some machine. She sees that her doctor is typing her information into the computer and she is fine with that. She is sure her doctor uses her information appropriately but hasn’t really thought much about it. She sees her home phone as the best way to access her healthcare providers, not some fancy technology. She feels technology is rather scary and it is too difficult for people like her.

**Site 3 Cluster D (Michael) – Good digital skills but concern over privacy and poor experience with digital services**

| Number of respondents | % in sample | Mean age | 1. Using technology to process health information | 2. Understanding of  health concepts and language | 3. Ability to actively  engage with digital services | 4. Feel safe and  in control | 5. Motivated to engage with digital services | 6. Access to digital  services that work | 7. Digital services  that suit individual needs |
| --- | --- | --- | --- | --- | --- | --- | --- | --- | --- |

| 6 | 5.4 | 52.0 | 2.73 | 3.17 | 3.48 | 2.27 | 2.73 | 2.41 | 2.14 |
| --- | --- | --- | --- | --- | --- | --- | --- | --- | --- |

Michael, 55, has a degree in Agriculture and runs a farm with his wife. He is a very organized person and keeps meticulous documentation of all his business records on the computer. He even scans his medical records and save them on his home computer.

He was recently told he has pre-diabetes and his doctor suggested changing his lifestyle to reduce the risk of developing type 2 diabetes. Since then, he has searched for diabetes information online and finds some of the online information useful. However, he always checks with his doctor to make sure he is getting the right and appropriate information.

He recently checked out the website of a local dietitian and hoped to make an appointment online. However, the booking only worked for return clients and he needed to call the practice as a new client. He knows there are fitness apps that he can use on his smartphone. However, he doesn’t like using a small screen and doesn’t really see the value of using these apps. The location tracking function on some of these apps also have him concerned about his privacy.

**Site 3 Cluster E (Mary) – Average digital skills with limited access to suitable digital services**

| Number of respondents | % in sample | Mean age | 1. Using technology to process health information | 2. Understanding of  health concepts and language | 3. Ability to actively  engage with digital services | 4. Feel safe and  in control | 5. Motivated to engage with digital services | 6. Access to digital  services that work | 7. Digital services  that suit individual needs |
| --- | --- | --- | --- | --- | --- | --- | --- | --- | --- |

| 35 | 31.3 | 56.9 | 2.48 | 2.82 | 2.55 | 3.03 | 2.49 | 2.66 | 2.32 |
| --- | --- | --- | --- | --- | --- | --- | --- | --- | --- |

After secondary school, Mary worked as a personal assistance but decided to move back to the country following her divorce ten years ago and helps out at her brother’s small organic farm. Now, 62, she lives alone but belongs to several local groups and has an active social calendar.

With the recent dry spell, Mary is feeling stressed about the farm. She uses the internet to search for strategies for coping with stress. While some of the information seem to be useful, some are confusing and some of those websites are difficult to navigate. So, she decides that her doctor is still her best source of health information.

She knows that computer systems can be hacked but believes that her digital health record is probably safe. After all, she doesn’t see why someone would want to see her records. The main use of her computer is to look for information and use emails to contact members of her social groups. She is not really keen on using the internet because there is no broadband connection to her property and using satellite means slow connection and constant dropping out, both are very frustrating.

**Site 3 Cluster F (Lisa) – Average digital skills with poor access to suitable digital services**

| Number of respondents | % in sample | Mean age | 1. Using technology to process health information | 2. Understanding of  health concepts and language | 3. Ability to actively  engage with digital services | 4. Feel safe and  in control | 5. Motivated to engage with digital services | 6. Access to digital  services that work | 7. Digital services  that suit individual needs |
| --- | --- | --- | --- | --- | --- | --- | --- | --- | --- |

| 13 | 11.6 | 47.0 | 2.52 | 2.85 | 2.46 | 2.48 | 2.49 | 2.19 | 2.08 |
| --- | --- | --- | --- | --- | --- | --- | --- | --- | --- |

At 58, Lisa and her husband run a small café which can be very busy on the weekend. Her son is away studying at Melbourne. She has arthritis and so far, the medication is working fine.

She has a computer at home that is mainly used by her husband. She has a mobile phone and an iPad which she uses to take pictures and gets on Facebook to connect with family and friends. She tries looking up information about arthritis on the internet, but she is totally confused with all these types of arthritis and she honestly has no idea what type she’s having.

She has private health insurance but finds their website difficult to get what she needs and prefers to call or goes to the branch if she has questions. Her doctor’s practice doesn’t have online booking service and she feels fine as she thinks calling is so much easier. She has heard about the My Health Record and has checked out the website, but she has no idea where to locate her record and not even sure if it’s such a good idea to have her record being kept there.

**Site 3 Cluster G (Nancy) – Limited digital skills but think eHealth is fine**

| Number of respondents | % in sample | Mean age | 1. Using technology to process health information | 2. Understanding of  health concepts and language | 3. Ability to actively  engage with digital services | 4. Feel safe and  in control | 5. Motivated to engage with digital services | 6. Access to digital  services that work | 7. Digital services  that suit individual needs |
| --- | --- | --- | --- | --- | --- | --- | --- | --- | --- |

| 10 | 8.9 | 59.2 | 2.25 | 2.26 | 2.09 | 2.75 | 2.06 | 2.23 | 1.93 |
| --- | --- | --- | --- | --- | --- | --- | --- | --- | --- |

Nancy is 68 and works as a part-time cashier at the local supermarket while her husband is retired. She has been having some knee problems in the last few years but thinks it is just part of growing old.

She has a mobile phone and reads text messages, but she never replies as she is not sure how to do it. Her daughter gave her an iPad and downloaded a few card games for her. She enjoys playing those games, but always worries that she may mess up the iPad by touching the wrong icons. She knows that you can find health information on the internet, but she doesn’t see the need to do so. She also hears about health apps but has no idea what they are and where to find them.

For her, technology doesn’t have anything to do with her health. If she is not feeling well, she just goes to her doctor. Her doctor told her about something called the My Health Record in her last visit and she thought it sounded like a good idea. However, she doesn’t really sure what it is or how it works.

**Site 3 Cluster H (Robert) – Limited digital access and skills but good understanding of health**

| Number of respondents | % in sample | Mean age | 1. Using technology to process health information | 2. Understanding of  health concepts and language | 3. Ability to actively  engage with digital services | 4. Feel safe and  in control | 5. Motivated to engage with digital services | 6. Access to digital  services that work | 7. Digital services  that suit individual needs |
| --- | --- | --- | --- | --- | --- | --- | --- | --- | --- |

| 4 | 3.6 | 56.3 | 1.25 | 2.75 | 1.20 | 2.45 | 1.70 | 2.17 | 1.00 |
| --- | --- | --- | --- | --- | --- | --- | --- | --- | --- |

Robert, a carpenter before he retired, just turned 80 last month and is generally in good health apart from having high blood pressure and some eyesight problem. He takes his medication regularly and tries to walk with his wife at least 30 minutes every day.

While he has a mobile phone, his eyesight problem means it is difficult for him to read any of the text messages. Furthermore, he lives in an area with no internet access and the signal to his mobile phone is unreliable.

To him, technology is some new stuff that is difficult for him to learn and he has no intention of learning at this age. He is aware of computer hacking or misuse of users’ data from the news, that kind of reassures him that he’s better off without technology. He doesn’t think technology can help take care of his health. It is the doctor who can help. After all, he’s been living without this new stuff all his life and he is doing fine. So, why bothers when he’s going to die soon.
