# Supplementary material for: Co-designing eHealth and Equity Solutions: Application of the Ophelia (Optimizing Health Literacy and Access) Process
Source: Front Public Health. 2020 Nov 20;8:604401. doi: 10.3389/fpubh.2020.604401 (PMC7718029; doi:10.3389/fpubh.2020.604401)
Supplement: Supplementary file 4 [file Table_4.DOCX]

**Supplementary Material 4 - Thematic analysis of solutions from Site 1 co-design workshops by strategies**

| **Intervention/Solution** | | **Participant quote*** | **Main problem identified** | **Intervention level** | **Raised for vignette (cluster)** | **Raised at workshop*** |
| --- | --- | --- | --- | --- | --- | --- |
| **Strategy 1 – Provide training or encourage use of technologies** | | | | | | |
| 1 | Organize ways to assist patients to access the My Health Record^#^ (e.g., workshops) | *‘Maybe run a workshop here, for those who want to learn to use the My Health Record, mainly to understand, have an introduction and what’s happening and what’s gonna be uploaded and what wasn’t.’ – CM workshop* | Lack of digital skills | Individual | Michael (D) | CM & HP |
| 2 | Start with simple engagement to encourage clients to use technology such as receiving SMS to confirm appointments | *‘You can start from the basic level, such as SMS to confirm appointments, just to engage the patient with technology.’ – CM workshop* | Lack of digital skills | Individual | Michael (D) | CM |
| 3 | Support clients to choose appropriate digital device | *‘And there are some [phones] out there that has big screen to help, getting a phone that she can handle.’ – CM workshop* | Using inappropriate digital devices | Individual | Maria (G) | CM & HP |
| 4 | Ask client what level of digital information they want (e.g., starting with SMS) | *‘Ask the patient if he’s interested in receiving SMS or personal call.’ – CM workshop* | Lack of digital skills | Individual | Michael (D) | CM |
| 5 | Send small bites of information to client through technology, such as SMS, to encourage clients to use technology | *‘Just little thing, you can SMS, just to get them to use their phones.’ – CM workshop* | Lack of digital skills | Individual | Michael (D) | CM |
| 6 | Provide a ‘digital navigator’ to interact with clients in the waiting room to provide information or assist in using digital devices | *‘It would be good if the government can put out some funding and you got a digital person that you need, doing a one-on-one chat in the waiting room.’ – CM workshop* | Lack of digital skills | Policy | Anthony (F) | CM |
| 7^ | Ensure young tech savvy staff are on the clinical team to support clients to engage with technology | *‘What I’m trying to do is to have younger staff who is interested in this area to help patients get onto these devices… the idea being that they are savvy and can explain all these to the people.’ – HP workshop* | Lack of digital skills | Policy | Anthony (F) | HP |
| **Strategy 2 – Provide access to reliable and trustworthy eHealth resources** | | | | | | |
| 8 | Provide clients with a list of secure and reliable eHealth resources | *‘Offer pooled resources with links to credible health information websites’ – HP workshop* | Lack of access to credible and reliable online health resources | Individual | Jennifer (D)/  Michael (D) | CM & HP |
| 9^ | Support clients to choose appropriate eHealth resources such as health apps | *‘Can encourage him something like these sleep apps, anxiety reducing apps, that he can download, just an app, and they can be useful to combat his anxiety.’ – CM workshop* | Lack of access to credible and reliable online health resources | Individual | Anthony (F) | CM & HP |
| 10^ | Establish a way that the clinic’s recommended digital services and apps can be easily downloaded by clients to their own devices | *‘If we have on our website, that would be good, that’s all archived, they [patients] can download onto their own apps or their own devices.’ – HP workshop* | Lack of access to credible and reliable online health resources | Policy | Jennifer (D)/  Michael (D) | HP |
| **Strategy 3 – Support clients with concerns on privacy and security of eHealth systems** | | | | | | |
| 11 | Encourage social engagement through access to community services to build trust | *‘This is the community center that is nearest to you, they have migrant services, you can go visit them, and go to their website.’ – CM workshop* | Concern about internet security | Individual | Anthony (F) | CM |
| 12 | Educate clients on how eHealth services are provided with security and privacy considerations | *‘I don’t think people really understand that there is kind of a level of security that is required in order to download clinical information… probably might be interested to look at how my information is stored when I see someone, what’s the level of security.’ – CM workshop* | Concern about internet security | Individual | Anthony (F) | CM |
| 13 | Build trust through direct contact with an informed person/ professional who can recommend reliable information and services | *‘I think [human] contact is very important, someone recommended it, he trust the person.’ – CM workshop* | Concern about internet security | Individual | Anthony (F) | CM |
| **Strategy 4 – Ensure effective communication to meet individual needs** | | | | | | |
| 14 | Provide specific health information in convenient physical handouts (e.g., diet) | *‘Give patients physical handouts… it is direct and there is no interpretation, physical form manifest that you can take it with you.’ – HP workshop* | Inadequate understanding of own health condition | Individual | Michael (D)/  Maria (G) | HP |
| 15 | Encourage health professionals to understand clients’ needs (e.g., why they don’t want to monitor their own health) | *‘Need to know as to why they do not want to track their health’ – CM workshop* | Inadequate understanding of own health condition | Practitioner | Jennifer (D)/  Anthony (F) | CM |
| 16 | Include questions about reasons for making medical appointment when booking online for efficient consultation | *‘When you make your appointment, you fill in a questionnaire, what the appointment is about, what are the issues… all of that information could be there when you go into the appointment and save time.’ – CM workshop* | General discussion | Policy | General discussion | CM |
| 17^ | Ensure the data collected at online booking are available at the patients’ appointment | *‘Make sure the information provided in your online booking is also available to your doctors and they have the information they actually need to look after you.’ – CM workshop* | General discussion | Policy | General discussion | CM |
| **Strategy 5 – Harness family and social support** | | | | | | |
| 18 | Reduce social isolation through referral to social services | *‘Maybe linking him to other people who are in the same situation, other immigrants who are having the same issue.’ – CM workshop* | Lack of social support | Individual | Jennifer (D)/  Anthony (F)/  Maria (G) | CM & HP |
| 19 | Encourage family members or friends to help set up the My Health Record^#^ | *‘If he doesn’t want someone to know he has something, then, find a good friend who will show you how to just delete that little bit and then your health record is there.’ – CM workshop* | Lack of digital skills | Family | Michael (D) | CM |
| 20^ | Encourage and support family members to manage health for the elderly | *‘If I’m planning for a flyer or more sophisticated eHealth device… I’d say [to the family member, e.g.] do you mind write down their sugar level and when they go home, [support] whatever we teach Maria to do.’ – HP workshop* | Inadequate understanding of own health condition | Family | Maria (G) | CM & HP |
| 21 | Provide a space and opportunities for social networking among clients to share good health information | *‘We need some chairs, a little café out at the front and people can chat.’ – CM workshop* | Lack of social support | Policy | Maria (G) | CM |
| **Strategy 6 – Motivate clients to actively engage with own health** | | | | | | |
| 22 | Encourage clients to use technology and regular engagement with health professionals as incentives to monitor own health | *‘Maybe things to motivate her, things like Fitbit, count her steps, monitor her weight on that, can chart her progress. And then regular checks with the doctor is a good thing as well… it sort of keeps them motivated, keeps them engaged.’ – HP workshop* | Inadequate understanding of own health condition | Individual | Jennifer (D) | CM & HP |
| 23^ | Provide specific practical information that clients need while in the clinic | *‘Her family doctor has to get her the information, information like you know the NDIS.’ – CM workshop* | Inadequate understanding of own health condition | Individual | Jennifer (D)/  Anthony (F)/  Maria (G) | CM & HP |
| 24^ | Set up regular monitoring of health condition (e.g., blood glucose, diet, exercise) | *‘She needs regular glucose monitoring.’ – HP workshop* | Inadequate understanding of own health condition | Individual | Jennifer (D)/  Anthony (F)/  Maria (G) | CM & HP |
| 25 | Set up regular counselling about key risk factors (e.g., weight loss) | *‘She needs some counselling regarding dietary program, some exercises, and discussion about weight loss.’ – HP workshop* | Inadequate understanding of own health condition | Individual | Jennifer (D)/  Anthony (F)/  Maria (G) | CM & HP |
| 26 | Use social media to inform clients of health-promoting resources | *‘Place information on Facebook for patients, things like workshops coming up, or information on healthy week that you can share… for patients to read about.’ – CM workshop* | Inadequate understanding of own health condition | Policy | Jennifer (D) | CM |
| 27 | Work with local Municipal Councils which have a range of health promoting and wellbeing programs | *‘The best way, to get your message out there, is to send people to the council where they are already running the program… The council has similar things, [like] the walking clubs…’ – CM workshop* | Inadequate understanding of own health condition | Policy | Jennifer (D)/  Maria (G) | CM |
| **Strategy 7 – Use a tailored and multi-disciplinary approach to healthcare** | | | | | | |
| 28^ | Refer clients to health or social support services | *‘Her family doctor’s going to refer her to, you know, community groups, the psychologists or refer her to health and wellbeing programs.’ – CM workshop* | Lack of social support | Individual | Jennifer (D)/  Anthony (F)/  Maria (G) | CM & HP |
| 29 | Provide a comprehensive multi-disciplinary ‘one-stop-shop’ in one session with content that really helps clients | *‘There’s something we do, obviously, is a multi-disciplinary set up, like LIFE program, where a patient can see the doctor, see the dietitian, see the exercise physiologist, if they can do that in a one-stop shop, in one session, that really helps them.’ – HP workshop* | Inadequate understanding of own health condition | Policy | Jennifer (D)/  Anthony (F)/  Maria (G) | HP |
| **Strategy 8 – Build capacity for effective practice** | | | | | | |
| 30 | Ensure health professionals have a genuine understanding of available health education courses | *‘…not just saying there’s a workshop and you go because you are diabetic, but has the doctor done the workshop, do they know what they are actually asking their patients to? I think that’s important, walk the talk.’ – CM workshop* | Inadequate understanding of own health condition | Practitioner | Jennifer (D) | CM |
| **Strategy 9 – Provide access to conventional and digital health services** | | | | | | |
| 31^ | Provide clients with summaries of medical history and/or medication in printed formats | *‘I print out a summary of their medications, every time they come in, I’ll give it to them and ask if this is correct and we check it, and they got their own copy… if they go to the hospital, they have a summary.’ – HP workshop* | Inadequate understanding of own health condition | Policy | Michael (D)/  Maria (G) | HP |
| 32 | Build awareness among older clients that they don’t have to use online appointment booking (same day appointments can be made using the phone) | *‘There are certain emergency spots available every day that won’t get opened until the clinic opens, so, they can ring, and they need to know that they can ring at 8 o’clock in the morning and make appointment and get an emergency spot.’ – HP workshop* | Lack of digital skills | Policy | Maria (G) | HP |

*CM = community member, HP = health professional

^#^My Health Record – a personal electronic health record in Australia

^Ideas rated very important or essential by all rating questionnaire respondents
