# Supplementary material for: Co-designing eHealth and Equity Solutions: Application of the Ophelia (Optimizing Health Literacy and Access) Process
Source: Front Public Health. 2020 Nov 20;8:604401. doi: 10.3389/fpubh.2020.604401 (PMC7718029; doi:10.3389/fpubh.2020.604401)
Supplement: Supplementary file 5 [file Table_5.DOCX]

**Supplementary Material 5 - Thematic analysis of solutions from Site 2 co-design workshops by strategies**

| **Intervention/Solution** | | **Participant quote*** | **Issues identified** | **Intervention level** | **Raised for vignette (cluster)** | **Raised at workshop*** |
| --- | --- | --- | --- | --- | --- | --- |
| **Strategy 1 – Provide training or encourage use of technologies** | | | | | | |
| 1 | Advertise or provide access to technology training programs or technology access available from local government or community service groups | *‘When you go into a doctor’s surgery, I find it the information bulletin is mainly about support groups, for you know, all sorts of these diseases, and there’s no actual display board that says go to your library and learn to use a computer, you know, contact your local primary school they got a group of kids that teaches you to do ABCD.’ – CM workshop*  *‘Linking her to local services that can assist her with digital skills such as neighbor houses, senior citizen clubs and facilitate peer education to upskill her.’ – HP workshop* | Lack of digital skills | Individual | Barbara (E)/  Doris (I) | CM & HP |
| 2 | Provide simple written information on how to use digital devices | *‘If she’s shown the basic steps to communicate and have it in a written form in Greek so that she can use it.’ – CM workshop* | Lack of digital skills | Individual | Anna (H) | CM |
| 3 | Teach clients to use voice activated digital assistants such as Siri or accessibility features such as enlarged fonts | *‘There’s speech feature, she could use speech to text.’ – HP workshop* | Lack of digital skills | Individual | Anna (H) | HP |
| 4 | Support clients to choose appropriate digital device(s) | *‘It’s a wrong choice of technology, wrong equipment, [he needs] something user friendly, with a large text.’ – CM workshop* | Using inappropriate digital devices | Individual | James (G) | CM & HP |
| **Strategy 2 – Provide access to reliable and trustworthy eHealth resources** | | | | | | |
| 5^ | Provide clients with secure, reliable and culturally appropriate eHealth resources | ‘A site that will give the right information, cos I got on the internet and then being given ten different opinions.’ – CM workshop  *‘You guide them to a culturally appropriate site, a secure site that is best practice… to the ones to avoid, cos there are just too much information out there, that isn’t accurate.’ – HP workshop* | Lack of access to credible and reliable online health resources | Individual | Barbara (E)/  Anna (H)/  David (D)/  Ming (F) | CM & HP |
| 6 | Support clients to choose appropriate eHealth resources such as health apps | *‘I say one of the concerns is the misuse of the fitness apps that I have people downloaded apps and they don’t know how to use it properly.’ – HP workshop* | Lack of access to credible and reliable online health resources | Individual | David (D) | HP |
| 7 | Sharing of consumer-focused eHealth resources between partner organizations | *‘So, organizations sharing those information will be good… which are the secure sites to recommend to people.’ – HP workshop* | Lack of access to credible and reliable online health resources | Policy | Ming (F) | HP |
| **Strategy 3 – Support clients with concerns on privacy and security of eHealth systems** | | | | | | |
| 8 | Educate clients on how eHealth and telehealth services are provided with privacy consideration such as using presentations from security experts | *‘As an organization, how can we calm him, giving him confidence that this eHealth privacy is protected in the eHealth system.’ – HP workshop* | Concern about internet security | Individual | David (D) | HP |
| 9 | Provide demonstration video on website to show how to set up privacy setting | *‘What about this age group producing a web where they air this program, where somebody actually sits there and talks about privacy, and shows you how to change it.’ – CM workshop* | Concern about internet security | Individual | David (D) | CM |
| 10 | Advocate government to take responsibility in ensuring the safety and security of electronic health records | *‘I think the government does have a responsibility for the safety of health records to make them as secure as possible.’ – CM workshop* | Concern about internet security | Policy | David (D) | CM |
| 11 | Advocate for legislation on who can have access to personal digital health records | *‘The law has to keep up with regard to who and how they get the information.’ – CM workshop* | Concern about internet security | Policy | David (D) | CM |
| 12 | Encourage clients to carry notebook with personal medical history and medication if decide to opt out of the My Health Record^#^ | *‘You can speak to your family doctor that I don’t want that to put up… he carries something with him, I’m on this medication and that would solve the problem.’ – CM workshop* | Concern about internet security | Individual | David (D) | CM |
| **Strategy 4 – Provide technologies and eHealth systems that meet different needs** | | | | | | |
| 13 | Support the government to better understand community needs when using the My Health Record^#^ or myGov^#^ | *‘Why don’t they work with local community organizations and promote it at the same time, at the ground level, yes, we gonna do ABCD, what is your community need.’ – CM workshop* | Difficult-to-use eHealth systems | Policy | Anna (H) | CM |
| 14^ | Involve users when developing websites or digital technologies to match their needs and skills | *‘Those people who produce this stuff need to get out to the community, people who developed the websites need to have more idea of what is easy to use.’ – CM workshop* | Difficult-to-use eHealth systems | Policy | Anna (H) | CM |
| 15^ | Ensure organization information technology systems are working smoothly in order to work with clients efficiently | *‘We need computers that work well, iPads, mobile phones that connect to the internet, WiFi, so that we could use it for all our clients.’ – HP workshop* | General discussion | Policy | General discussion | HP |
| **Strategy 5 – Ensure effective communication to meet individual needs** | | | | | | |
| 16^ | Provide health information in multiple formats such as print, audio, video, diagrams, large print or appropriate languages | *‘Information should be easily available, and in multiple formats.’ – CM workshop*  *‘And I think he is more a visual person, he enjoys seeing the picture, so something, that is more graphic, rather than text, would be more appropriate for him.’ – HP workshop* | Inadequate understanding of own health condition/  Language barrier | Individual | Barbara (E)/  Anna (H)/  James (G)/  Ming (F) | CM & HP |
| 17^ | Translate health information into appropriate languages as well as present culturally appropriate advice and recommendations on website | *‘I think it’s also the provision of culturally appropriate advice and recommendations because, for example, on the Australian website, the advice of, the information is based on, for example, for cholesterol, it’s based on the western diet, so, if he is having a Chinese diet, I don’t think he can relate to that information.’ – HP workshop* | Language barrier | Individual | Ming (F) | CM & HP |
| 18^ | Encourage clinicians to listen to clients’ needs and take different approaches to engage instead of a one-size-fits-it-all approach | *‘Don’t assume, that client-centered self-management type of approach is going to be one-size-fits-it-all and it’s not appropriate in many instances such as for the Chinese communities, so, I guess we just need to keep being aware that there are other ways of engaging with people.’ – HP workshop* | Inadequate understanding of own health condition | Practitioner | Ming (F) | HP |
| 19^ | Encourage clinicians to use plain language and write down information and instructions for clients | *‘It should be a standard ‘would you like me to write it down for you’.’ – CM workshop*  *‘You gotta say a common thing, we would say belly instead of abdomen, so, she has to be up to that for them to understand.’ – CM workshop* | Inadequate understanding of own health condition | Practitioner | Anna (H) | CM |
| 20^ | Support practitioners with access to culturally appropriate resources | *‘Access to culturally appropriate resources for health professionals who can tailor culturally appropriate advice to patients.’ – HP workshop* | Inadequate understanding of own health condition | Practitioner | Ming (F) | HP |
| 21 | Allow for flexibility in appointment duration to ensure clients’ needs are met | *‘Let them know I need a long appointment.’– CM workshop* | Inadequate understanding of own health condition | Policy | Barbara (E) | CM |
| 22^ | Ensure interpreters are available for culturally and linguistic diverse communities | *‘If he comes into this building, we need to make sure that we have an interpreter and interpret what his needs are in his own language because some of people who speaks his language, their English is quite good but they don’t always understand what the clinician might be telling them and, likewise, the clinician might not always understand what their needs are.’ – HP workshop* | Language barrier | Policy | Ming (F) | CM & HP |
| 23^ | Establish a multicultural workforce to meet the different needs of culturally and linguistic diverse communities | *‘We have people who may be a better fit for certain clients, because they do come from an Asian background which may just start to break down some of the barriers of like, okay, this is someone who is going to understand what I do basically.’ – HP workshop* | Language barrier | Policy | Ming (F) | CM & HP |
| **Strategy 6 – Harness family and social support** | | | | | | |
| 24 | Encourage volunteers, friends or family members to provide regular practice in using technologies through one-on-one coaching or mentoring | *‘I used to get her to text me, text me ‘hello’ and I’ll text you back, I wanted that to come through every single day. And she was into this routine and we added a few extra words and now she is fine.’ – CM workshop*  *‘My mother is learning from her grandkids.’ – HP workshop* | Lack of digital skills | Family/  Social | Barbara (E)/  Anna (H) | CM |
| 25 | Provide older clients with access to age-specific technology club | *‘Why not start a technology club for people of a certain age groups, without being detrimental, they would be in a group that they are comfortable in.’ – CM workshop* | Lack of digital skills | Family/  Social | Anna (H) | CM |
| 26 | Encourage family member or carer to set up the My Health Record with preferred privacy setting for older clients | *‘Somebody just to go through and set up My health record to the way that she would like.’ – CM workshop* | Lack of digital skills | Family | Doris (I) | CM |
| 27^ | Provide family members with appropriate health information | *‘Maybe invite the wife to the heart health program so that they are all on the same page and get the same information.’ – HP workshop* | Inadequate understanding of own health condition | Family | James (G) | HP |
| 28 | Encourage family member or carer to accompany clients from culturally and linguistic diverse communities to consultations even if interpreter is available | *‘She may need to take one of her children or somebody with her that speaks Greek to go with her to the doctor.’ – CM workshop*  *‘Offer her interpreters even with the presence of family members.’ – HP workshop* | Language barrier | Family | Anna (H) | CM & HP |
| 29 | Work with local schools to encourage children to train the elderly to use technologies | *‘I remember myself in year 7, I used to go to a nursing home and read as volunteer to help, so, I’m sure they would be able to explain, the basic.’ – CM workshop* | Lack of digital skills | Family/  Social | Barbara (E) | CM |
| 30 | Support family in caring for clients such as organizing cooking class for couples | *‘Their idea of healthy may not be actually healthy… you know, just their idea of healthy, sometimes, just isn’t healthy. So, maybe have his wife involved with a dietician, healthy cooking classes for two people, something like that.’ – HP workshop* | Inadequate understanding of own health condition | Policy | James (G) | HP |
| 31 | Work with local ethnic community organizations to promote health and wellbeing among culturally and linguistic diverse communities | *‘Maybe her doctor needs to, or her medical center needs to access one of the community groups, get that medical information out there.’ – CM workshop* | Inadequate understanding of own health condition | Policy | Anna (H) | CM |
| 32 | Provide access to local support services for clients from culturally and linguistic diverse communities | *‘Actually, they have a refugee support group in, actually in this building as well, for people with special needs.’ – CM workshop* | Lack of social support | Policy | Ming (F) | CM |
| **Strategy 7 – Motivate clients to actively engage with own health** | | | | | | |
| 33 | Educate clients about eHealth and how it may relate to their health condition | *‘I think she needs to be educated as to what is out there.’ – CM workshop* | Lack of digital skills | Individual | Ming (F)/  Doris (I) | CM & HP |
| 34 | Connect clients’ interest to technologies and provide positive experiences such as using iPad to demonstrate exercise or provide feedback during group activity sessions or consultations | *‘While she’s in the gym or in consultation, there are opportunities to give her some positive experience of technology, watching how exercise is being done or feedback on how she performs, give her some positive experiences about technology… And if she has some questions, we can show her the answer on iPad.’ – HP workshop* | Lack of motivation to use technology | Individual | Doris (I) | HP |
| 35^ | Educate clients about their health conditions, assist them to set up personal goals and link their interest to health-promoting activities | *‘He needs to be involved in setting his own goal.’ – HP workshop*  *‘Encourage him to do the things he loves, he loves nature, going for a walk in the bush or the beach, and don’t sit there drinking.’ – HP workshop* | Inadequate understanding of own health condition | Individual | James (G) | HP |
| **Strategy 8 – Use a tailored and multi-disciplinary approach to healthcare** | | | | | | |
| 36^ | Refer clients to other related health support services that are beneficial to their health conditions | *‘Are his feet contributing to his knee problem? Could refer him to a podiatry review, refer to other allied health professionals.’ – HP workshop* | Inadequate understanding of own health condition | Individual | Barbara (E)/  Ming (F)/  David (D) | CM & HP |
| 37^ | Support clinicians with better access to medical history of clients (with clients’ consent) to facilitate a team-approach to healthcare | *‘And I wonder if there is a link in there in our organization to, could be viewing his medical information more effectively, so making sure that we are making safe and relevant recommendations for him with his consent.’ – HP workshop* | Inadequate understanding of own health condition | Policy | James (G) | HP |
| **Strategy 9 – Build capacity for evidence-based practice** | | | | | | |
| 38^ | Provide clinicians with ongoing professional development on eHealth | *‘Seriously, we need to know eHealth a lot better so that we can actually give confidence to our patients that their privacy is protected.’ – HP workshop* | Lack of digital skills | Practitioner | Ming (F) | HP |
| 39^ | Explore best practice and health evidence relevant to other cultures and support clinicians with ongoing professional development on cultural diversity | *‘Explore the evidence, the health evidence from different cultures, and not just translate western guidelines.’ – HP workshop* | Inadequate understanding of own health condition | Practitioner | Ming (F) | HP |
| **Strategy 10 – Provide access to conventional and digital health services** | | | | | | |
| 40 | Offer services such as exercise training over Skype to establish regular contact with clients and encourage technology use | *‘Using Skype for training, reinforcing, more regular contact.’ – HP workshop* | Lack of motivation to use technology | Policy | Anna (H) | HP |
| 41^ | Provide home visit services to assist patients with healthcare management | *‘Maybe provide help with exercise program in the home.’ – HP workshop* | Inadequate understanding of own health condition | Policy | Anna (H) | HP |
| 42 | Offer or referral of after-hours health services for people working full time | *‘They have programs like tai chi and meditations… A lot of them have after hours.’ – CM workshop* | Inadequate understanding of own health condition | Policy | David (D) | CM & HP |
| 43^ | Keep in mind that there are people who are ‘out of the web’ in strategic planning | *‘If you push her and use technology, she may lose all that confidence she’s got… Organizations need to keep in mind that there are people who are out of the web.’ – CM workshop* | Lack of digital skills | Policy | Doris (I) | CM |

*CM – community member, HP – health professional

^#^My Health Record – a personal electronic health record in Australia; myGov – the Australian online portal to access government services

^Ideas rated very important or essential by all rating questionnaire respondents
