# Supplementary material for: Co-designing eHealth and Equity Solutions: Application of the Ophelia (Optimizing Health Literacy and Access) Process
Source: Front Public Health. 2020 Nov 20;8:604401. doi: 10.3389/fpubh.2020.604401 (PMC7718029; doi:10.3389/fpubh.2020.604401)
Supplement: Supplementary file 6 [file Table_6.DOCX]

**Supplementary Material 6 - Thematic analysis of solutions from Site 3 co-design workshops by strategies**

| **Intervention/Solution** | | **Participant quote*** | **Issue identified** | **Intervention level** | **Raised for vignette (cluster)** | **Raised at workshop*** |
| --- | --- | --- | --- | --- | --- | --- |
| **Strategy 1 – Provide training or encourage use of technologies** | | | | | | |
| 1 | Support clients to choose or change to appropriate digital device(s) | *‘Maybe he needs a new device, it’s too small.’ – CM workshop* | Using inappropriate digital devices | Individual | Michael (D) | CM |
| 2 | Provide training to use eHealth resources such as accessing the My Health Record^#^ | *‘If somebody taught her, maybe she can use it.’ – CM workshop* | Lack of digital skills | Individual | Mary (E)/  Lisa (F)/  Nancy (G) | CM & HP |
| 3 | Inform clients about places in the community where they can access technology training programs (e.g., library/ community hub/ U3A^#^) | *‘Some maybe at community centers, groups, courses, for people who can actually improve, suggest them to join U3A.’ – HP workshop* | Lack of digital skills | Individual | Mary (E)/  Lisa (F)/  Nancy (G) | CM & HP |
| 4 | Have clinic staff show clients how to use the My Health Record^#^ or run information sessions | *‘Maybe the clinics can run an information session on how you can go into the My Health Record or can have a staff member to help people.’ – CM workshop* | Lack of digital skills | Individual | Mary (E)/  Lisa (F)/  Nancy (G) | CM & HP |
| 5 | Provide clients with computers in clinics and hospitals to use for health purposes | *‘Hospital or doctor surgery can make computer available, have staff, show people teach people how to access their information.’ – CM workshop* | No access to internet connection | Individual | Mary (E) | CM & HP |
| **Strategy 2 – Provide access to reliable and trustworthy eHealth resources** | | | | | | |
| 6 | Give clients specific links to navigate to appropriate websites | *‘If she’s having trouble navigating, you can actually show her how to find it, and give her the links.’ – HP workshop* | Lack of digital skills | Individual | Mary (E)/  Lisa (F)/  Nancy (G) | CM & HP |
| 7 | Support clients to choose appropriate eHealth resources such as health apps or emergency apps | *‘She can use emergency app, you don’t need connect, 000 still works even without signals, it’ll ping off the satellite.’ – CM workshop* | Lack of access to credible and reliable online health resources | Individual | Michael (D)/  Nancy (G)/  Robert (H)/  Mary (E) | CM & HP |
| 8 | Provide clients a list of secure and reliable eHealth resources | *‘You can refer her to the right arthritis specific website.’ – HP workshop* | Lack of access to credible and reliable online health resources | Individual | Mary (E)/  Lisa (F) | HP |
| 9 | Provide specific YouTube clips (visual/ verbal) as step by step guides | *‘Create YouTube things, step by step guide’ – CM workshop* | Lack of access to credible and reliable online health resources | Individual | Mary (E)/  Lisa (F)/  Nancy (G)/  Robert (H) | CM & HP |
| 10 | Doctors make specific recommendations about which software or apps to use | *‘And depends on her diagnosis, there might be apps, exercise apps that might help her, a website or YouTube clips.’ – HP workshop* | Lack of access to credible and reliable online health resources | Individual | Michael (D)/  Nancy (G)/  Robert (H)/  Mary (E) | CM & HP |
| **Strategy 3 – Support clients with concerns on privacy and security of eHealth systems** | | | | | | |
| 11 | Present the benefits of technology to encourage clients to take up technology for health | *‘Everyone is scared of technology, it’s about ensuring the positive side, it can protect them – CM workshop* | Concern over internet security | Individual | Nancy (G)/  Robert (H) | CM & HP |
| 12^ | Provide a health summary in physical form if decide to opt out of the My Health Record | *‘How about have this lovely [health] summary, in case he doesn’t want to be on the My Health Record.’ – HP workshop* | Concern over internet security | Individual | Robert (H) | HP |
| 13 | Provide forums where clients can discuss the features and benefits of the My Health Record^#^ | *‘Have a discussion about the My Health Record, how it benefits her, how she can put as much or as little as she wants.’ – HP workshop* | Concern over internet security | Individual | Lisa (F)/  Robert (H)/  Nancy (G) | HP |
| **Strategy 4 – Provide technologies and eHealth systems that meet different needs** | | | | | | |
| 14^ | Advocate government to ensure health records on the My Health Record^#^ are up to date | *‘Government needs to make the health record current, up to date’ – CM workshop* | Difficult-to-use eHealth systems | Policy | Lisa (F) | CM |
| **Strategy 5 – Ensure effective communication to meet individual needs** | | | | | | |
| 15 | Provide easy-to-understand health information in diverse formats (e.g., print, video, large prints etc.) | *‘You can give her information, I mean, face-to-face verbally, or give her pamphlets, brochures, make sure you have brochures, free ones.’ – HP workshop* | Inadequate understanding of own health condition | Individual | Mary (E)/  Lisa (F)/  Nancy (G)/  Robert (H) | CM & HP |
| 16 | Provide standards for staff producing written information (e.g., text size, minimal text) | *‘An important take home message is I use to type in a really big font, just very simple on a page, should educate staff to have very big fonts for older people.’ – HP workshop* | Inadequate understanding of own health condition | Policy | Mary (E)/  Lisa (F)/  Nancy (G)/  Robert (H) | CM & HP |
| **Strategy 6 – Harness family and social support** | | | | | | |
| 17 | Encourage volunteers, family members or carers to support the elderly in using technology for health | *‘And give something to her daughter to load it up for her, have the family to support.’ – HP workshop*  *‘Have family, friends, kids to set up health information and save it on their phone.’ – CM workshop* | Lack of digital skills | Family | Nancy (G)/  Robert (H) | CM & HP |
| **Strategy 7 – Motivate clients to actively engage with own health** | | | | | | |
| 18^ | Educate clients about their diagnosis and health conditions | *‘She needs some education around her diagnosis.’ – HP workshop* | Inadequate understanding of own health condition | Individual | Michael (D)/  Mary (E)/  Lisa (F)/  Nancy (G) | CM & HP |
| 19 | Provide access to community educators or nurses to promote understanding of own health condition | *‘I had diabetes, I was having trouble accessing information, I had some real issues, but I think there’s no support, there should more access to information.’ – CM workshop* | Inadequate understanding of own health condition | Individual | Michael (D) | CM |
| 20^ | Regular assessment/monitoring of clients with chronic conditions | *‘We can have regular assessment so that we can monitor his health.’ – HP workshop* | Inadequate understanding of own health condition | Individual | Robert (H) | HP |
| 21^ | Doctors to support clients to understand health information | *‘He needs some help with information from doctor.’ – CM workshop* | Inadequate understanding of own health condition | Individual | Michael (D)/  Mary (E)/  Lisa (F)/  Nancy (G) | CM & HP |
| 22^ | Help clients with decisions about what specific lifestyle changes are appropriate over time | *‘He needs to find out what sort of lifestyle changes, whether it is working or not, whether it’s exercise more or changing his diet, so that he can do the right thing himself.’ – CM workshop* | Inadequate understanding of own health condition | Individual | Michael (D)/  Mary (E)/  Lisa (F)/  Nancy (G) | CM & HP |
| 23 | Hold community events (field day, stall, free clinic etc.) on health and wellbeing and technology | *‘If you have public events, have a stall setup, people might pick up something.’ – CM workshop*  *‘Have a field day, free clinic’ – HP workshop* | Inadequate understanding of own health condition | Policy | Robert (H) | CM & HP |
| **Strategy 8 – Use a tailored and multi-disciplinary approach to healthcare** | | | | | | |
| 24 | Refer clients to support groups | *‘The doctor might need to refer him to some diabetes support groups.’ – CM workshop* | Inadequate understanding of own health condition | Individual | Michael (D)/  Mary (E)/  Nancy (G) | CM & HP |
| 25^ | Provide tailored health management plan | *‘She needs a management plan.’ – HP workshop* | Inadequate understanding of own health condition | Individual | Michael (D)/  Mary (E)/  Lisa (F)/  Nancy (G) | CM & HP |
| 26 | Refer clients to key services, e.g., mental health, exercise etc. | *‘She needs a referral to an exercise group.’ – HP workshop* | Inadequate understanding of own health condition | Individual | Michael (D)/  Mary (E)/  Nancy (G) | CM & HP |
| 27 | Ensure clients get support services in their homes/community between tests, diagnosis and treatments | *‘The time lapse, when go to the family doctor, get a referral, it’s months… you can have more community educators that we can go to them, as opposed to wait for these experts.’ – CM workshop* | Inadequate understanding of own health condition | Individual | Michael (D) | CM |
| 28 | Ensure clients know how to use information services such as Nurse-on-call | *‘I use Nurse-on-call, you can call them. It’s brilliant.’ – CM workshop* | Inadequate understanding of own health condition | Individual | Michael (D) | CM |
| **Strategy 9 – Build capacity for effective practice** | | | | | | |
| 29^ | Support health professionals with ongoing professional development on the use of the My Health Record^#^ | *‘Staff need to update their knowledge of the My Health Record.’ – HP workshop* | Lack of digital skills | Practitioner | Lisa (F) | HP |
| **Strategy 10 – Provide access to conventional and digital health services** | | | | | | |
| 30 | Connect with clients using appropriately tailored communication platform | *‘Don’t send him SMS, remind staff to call not text.’ – HP workshop* | Lack of digital skills | Policy | Robert (H) | HP |
| 31^ | Have paper-based brochures or fact sheets available for people who don’t use internet | *‘And she doesn’t want to look on the internet, we can provide her with brochures or fact sheets, paper-based.’ – HP workshop* | Lack of digital skills | Policy | Mary (E)/  Lisa (F)/  Nancy (G)/  Robert (H) | CM & HP |
| 32 | Ensure that there are mechanisms to support people who are not using technology | *‘I read somewhere that there are about 15% of people who don’t use the internet. All organizations must have some mechanism to make sure that this 15% is not being left behind.’ – CM workshop* | Lack of digital skills | Policy | Nancy (G) | CM |

*CM = community member, HP = health professional

^#^My Health Record – a personal electronic health record in Australia; U3A – University of the third age

^Ideas rated very important or essential by all ideas rating questionnaire respondents
