# Supplementary material for: Co-designing eHealth and Equity Solutions: Application of the Ophelia (Optimizing Health Literacy and Access) Process
Source: Front Public Health. 2020 Nov 20;8:604401. doi: 10.3389/fpubh.2020.604401 (PMC7718029; doi:10.3389/fpubh.2020.604401)
Supplement: Supplementary file 7 [file Table_7.DOCX]

**Supplementary Material 7 | Evidence of application of the OPtimizing HEalth LIteracy and Access (Ophelia) guiding principles***

| **Evidence** | **Limitations** | **Application** |
| --- | --- | --- |
| **P1. Outcome focused** |  |  |
| - The aim was to determine if the eHLQ could be used to understand eHealth literacy needs and help develop interventions aimed to improve health. | - Participating sites viewed the study as a pilot project and did not establish individual aims for improving health and equity outcomes. | Partially applied |
| **P2. Equity driven** |  |  |
| - Face-to-face interviews were available to ensure older people and people with lower literacy were included in the needs assessment survey. - The eHLQ cluster analysis process ensured that small groups facing eHealth literacy challenges, such as the single-member cluster at Site 1, were included. - Intervention ideas such as providing non-digital or culturally appropriate health information were co-designed to address the needs of people with limited eHealth access or migrants. | - Limited number of respondents for the semi-structured interviews, especially those most at-risk, to help better understand inequitable experiences. | Mostly applied |
| **P3. Co-design approach** |  |  |
| - Intervention ideas were generated through co-design workshops with community members and frontline health workers. | - Low participation rate for the workshops possibly because many viewed the workshops as a pilot project instead of an exercise for service and system improvement for their organizations. - Information technology professionals who could provide a digital perspective were not involved. | Mostly applied |
| **Evidence** | **Limitations** | **Application** |
| **P4. Needs-diagnostic approach** |  |  |
| - Using the eHLQ, the cluster analyses identified eight to nine client groups for the individual site with different eHealth literacy strengths and weaknesses. - By combining the cluster analyses with demographic and semi-structured interview data, local needs such as lack of internet infrastructure were identified. | - Specific data relevant to individual sites were not collected. - Limited number of respondents for the semi-structured interviews to help better understand local needs. | Mostly applied |
| **P5. Driven by local wisdom** |  |  |
| - Intervention ideas were generated based on the local experience and knowledge of community members and clinicians through the co-design workshops. | - Low participation rate for the workshops. | Mostly applied |
| **P6. Sustainable** |  |  |
| - Only involved Phase 1 of the Ophelia process. | - Evidence of sustainability remained to be collected. | Not determined |
| **P7. Responsiveness** |  |  |
| - By using the eHLQ combined with demographic and interview data, vignettes were developed, providing insights into the eHealth literacy challenges faced by different client groups. To respond to these challenges, a range of 32 to 43 ideas, targeted at the different needs of client groups, were generated at the sites. | - Specific data relevant to individual site were not collected. | Mostly applied |
| **P8. Systematically applied** |  |  |
| - The intervention ideas generated at all 3 sites encompassed the individual, family, practitioner and policy levels. | - Evidence on how application of the multilevel ideas could optimize eHealth literacy remained to be collected. | Partially applied |

* See Table 1 for a description of the Ophelia principles.
